# Extensive Diversity of Prion Strains Is Defined by Differential Chaperone Interactions and Distinct Amyloidogenic Regions

**DOI:** 10.1371/journal.pgen.1004337

**Published:** 2014-05-08

**Authors:** Kevin C. Stein, Heather L. True

**Affiliations:** Department of Cell Biology and Physiology, Washington University School of Medicine, St. Louis, Missouri, United States of America; Northwestern University, United States of America

## Abstract

Amyloidogenic proteins associated with a variety of unrelated diseases are typically capable of forming several distinct self-templating conformers. In prion diseases, these different structures, called prion strains (or variants), confer dramatic variation in disease pathology and transmission. Aggregate stability has been found to be a key determinant of the diverse pathological consequences of different prion strains. Yet, it remains largely unclear what other factors might account for the widespread phenotypic variation seen with aggregation-prone proteins. Here, we examined a set of yeast prion variants of the [*RNQ*+] prion that differ in their ability to induce the formation of another yeast prion called [*PSI*+]. Remarkably, we found that the [*RNQ*+] variants require different, non-contiguous regions of the Rnq1 protein for both prion propagation and [*PSI*+] induction. This included regions outside of the canonical prion-forming domain of Rnq1. Remarkably, such differences did not result in variation in aggregate stability. Our analysis also revealed a striking difference in the ability of these [*RNQ*+] variants to interact with the chaperone Sis1. Thus, our work shows that the differential influence of various amyloidogenic regions and interactions with host cofactors are critical determinants of the phenotypic consequences of distinct aggregate structures. This helps reveal the complex interdependent factors that influence how a particular amyloid structure may dictate disease pathology and progression.

## Author Summary

Protein conformational disorders, including many neurodegenerative diseases, result when a protein misfolds and undergoes a conformational change to form self-templating aggregates, called amyloid. Interestingly, the proteins that misfold in these diseases tend to form a wide variety of distinct aggregate structures. In prion diseases, these different amyloid conformations are called prion strains. The different conformations of prion strains are responsible for modulating disease progression, pathology, and transmission. Previous work with yeast prions has provided tremendous insight into how distinct prion conformers can cause such phenotypic variability. Here, we used a set of [*RNQ*+] prion variants to show the complex web of interactions involved in the propagation of distinct aggregate structures. We found that several different non-adjacent regions of Rnq1, even outside the prion-forming domain, make varying contributions to the propagation of distinct variants of the [*RNQ*+] prion. Moreover, our data support the hypothesis that the [*RNQ*+] variants differentially interact with the molecular chaperone Sis1. These data strongly suggest that the variable phenotypic manifestations of different aggregate conformations depend upon a unique set of primary structural elements and differential interactions with host cofactors.

## Introduction

The misfolding of proteins to form cross-β sheet amyloid structures is characteristic of a variety of diseases, including many neurodegenerative disorders, such as Alzheimer's disease [1]. Increasing evidence suggests that these protein conformational disorders are caused by a similar etiological mechanism, in which the amyloid that forms represents an infectious, self-templating structure that is often described as prion-like [2], [3]. Prion diseases are caused when the native form of the prion protein (PrP^C^) misfolds and aggregates to form the amyloid structure called PrP^Sc^ [4]. PrP^Sc^ is considered infectious because it transmits its pathogenic conformation by templating the conversion of other native PrP^C^ monomers to PrP^Sc^ in a self-propagating fashion [5].

To add another layer of complexity, it now appears that the proteins that misfold in these disorders can adopt an array of different aggregated conformations, called prion strains in prion diseases [6]–[8]. Prion strains often have unique biochemical properties and encode different degrees of infectivity [9]. These differences are thought to be the underlying cause of the widespread pathological variation seen in prion diseases. It was recently shown that distinct self-propagating structures of α-synuclein, the protein that misfolds in Parkinson's disease, also exist and promote the formation of tau inclusions to different extents [10]. Yet, with estimates that a single amyloidogenic protein like PrP may propagate over 30 distinct aggregate conformers [11], it is unclear what underlying factors contribute to such widespread structural and phenotypic diversity.

A tremendous amount of insight into the physical basis of prion strains, and amyloid polymorphism in general, has come from studying the prion strains that are endogenous to the budding yeast *Saccharomyces cerevisiae* (here also called prion variants). Like mammalian amyloidogenic proteins, yeast prion proteins form self-propagating, β sheet-rich amyloid structures. These self-perpetuating yeast prions exhibit dominant, non-Mendelian inheritance that promotes variation in cellular phenotypes [12]. One of the most well-studied yeast prions is the [*PSI*+] prion, formed from the translation termination factor Sup35. Sup35 is soluble and functional in [*psi*−] cells, but Sup35 misfolds and is sequestered into prion aggregates in [*PSI*+] cells, resulting in a nonsense suppression phenotype [13], [14]. Two [*PSI*+] prion variants were initially categorized based on the degree of nonsense suppression [15]. Cells propagating the strong [*PSI*+] variant showed increased nonsense suppression relative to cells propagating the weak [*PSI*+] variant. Yet, like prion strains of PrP^Sc^, it has become clear that a large continuum of different [*PSI*+] variants also exists [16]–[18].

Studies of conformationally distinct amyloid fibers of the prion-forming domain (PFD) of Sup35 formed *in vitro* have helped reveal a foundation to explain the structural and phenotypic differences between [*PSI*+] variants [19]–[23]. Like most yeast prions, and even several disease-associated proteins, Sup35 has a PFD that is rich in glutamine and asparagine (Q/N) residues, and is necessary for the maintenance of [*PSI*+] [12], [15], [24]–[27]. In distinguishing [*PSI*+] variants, it was shown that the contiguous length of the Sup35-PFD that forms the amyloid core correlates with a set of interdependent biochemical properties, including fiber stability, average aggregate size, and replication kinetics [19]–[21], [28]–[30]. Collectively, these parameters dictate a balance between fiber fragmentation and fiber growth, which is the major biophysical determinant of the resulting [*PSI*+] phenotype [20]. In the case of strong [*PSI*+] cells, the shorter and more fragile amyloid core of the Sup35 aggregates gives rise to more fragmentation and greater sequestration of Sup35 into aggregates, thereby resulting in more nonsense suppression. The more stable structure of Sup35 aggregates in weak [*PSI*+] cells, on the other hand, does not as readily fragment or capture monomer; hence, a larger pool of functional protein remains to participate in translation termination. These biochemical parameters have served as the foundation to describe the structural basis of prion strains, and have been shown to apply to various PrP^Sc^ strains [31], [32]. However, these correlations do not always hold true for the wide variety of structural variants that are possible for [*PSI*+], PrP^Sc^, and even another yeast prion, [*RNQ*+] [11], [33]–[37]. Thus, it remains a mystery what other variables help determine the extensive phenotypic differences exhibited by different prion strains.

Interestingly, while [*PSI*+] modulates translation termination, the major phenotypic manifestation of the [*RNQ*+] prion (also called [*PIN*+] and formed from the Rnq1 protein) is inducing the *de novo* formation of [*PSI*+] [38]–[43]. The formation of [*PSI*+], but not its continued propagation, relies on the presence of the [*RNQ*+] prion [38]. Five different variants of the [*RNQ*+] prion have been categorized based on how frequently they facilitate [*PSI*+] formation, from low rates to very high rates [44]. These [*RNQ*+] variants were also classified by the aggregate pattern observed in cells expressing GFP-tagged Rnq1 [45]. Cells predominately showing one fluorescent focus were called single-dot (s.d.) [*RNQ*+], while cells having many foci were called multiple-dot (m.d.) [*RNQ*+]. Recently, it was demonstrated that Rnq1 can form over 40 variants of the [*RNQ*+] prion [33], and different [*RNQ*+] variants were also found in wild yeast isolates [46]. While the mechanism of how [*RNQ*+] affects [*PSI*+] formation remains to be elucidated, a number of studies suggest that [*RNQ*+] acts as an imperfect template that interacts with Sup35 to cross-seed the induction of [*PSI*+] [47]–[49]. Indeed, similar to Sup35, Rnq1 has a Q/N-rich PFD that is necessary for prion propagation and may facilitate the interaction with Sup35 [42], [49], [50]. However, while some differences in biochemical and cellular properties have been noted between [*RNQ*+] variants, none of these properties explain how [*RNQ*+] variants differentially promote [*PSI*+] formation [33], [34], [44], [45], [51], [52].

Here, we set out to determine what factors distinguish [*RNQ*+] prion variants to allow for such dramatic phenotypic differences in [*PSI*+] inducibility. We found that each [*RNQ*+] variant relies on a distinct set of non-adjacent regions for both propagation and interaction with Sup35. While there is normally much emphasis on Q/N-rich prion-like domains, we show that regions outside of the canonical Rnq1-PFD influence [*RNQ*+] propagation in a variant-dependent manner. Furthermore, our data provide striking support for the hypothesis that different interactions between amyloid and molecular chaperones, and potentially other cellular cofactors, influence the phenotypic manifestations of distinct prion variants. This work helps reveal the structural and biological complexity underlying prion strains, showing that a large set of interdependent factors likely contribute to the ability of distinct aggregate conformations to modulate disease phenotype and transmissibility.

## Results

### Fiber fragmentation parameters do not distinguish [*RNQ*+] variants

**Figure 1:**
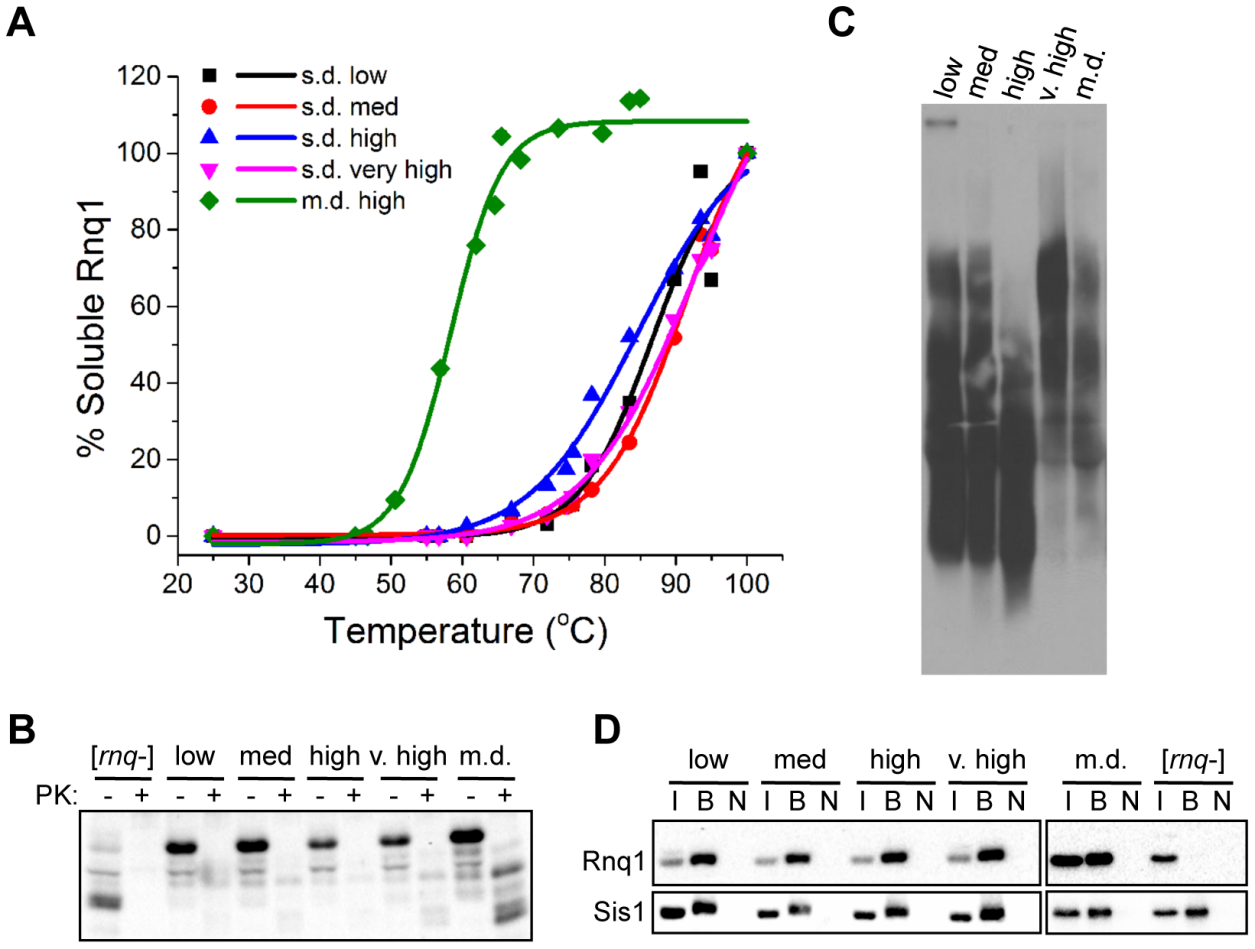
Biophysical parameters associated with fiber fragmentation do not distinguish [*RNQ*+] variants. (A) Rnq1 aggregates of s.d. [*RNQ*+] variants have similar thermal stability and are more stable than m.d. high [*RNQ*+]. Lysates were treated with a temperature gradient, followed by SDS-PAGE and western blot using an αRnq1 antibody. Rnq1 was quantified using ImageJ, normalized to the 100°C band, and plotted using Origin 9.0 software. Data are representative of at least five independent experiments. (B) Rnq1 aggregates of m.d. high [*RNQ*+] have increased protease resistance as compared to the s.d. [*RNQ*+] variants. Lysates of [*rnq*−] cells or cells propagating the indicated [*RNQ*+] variant were incubated with 2 µg/mL proteinase K (PK) at 37°C for 1 hr, followed by SDS-PAGE and western blot with an αRnq1 antibody. (C) Relative Rnq1 aggregate distribution for each [*RNQ*+] variant does not correlate to rate of [*PSI*+] induction. Lysates of yeast cells propagating s.d. low, s.d. medium, s.d. high, s.d. very high, and m.d. high (m.d.) [*RNQ*+] were subjected to SDD-AGE and western blot using an αRnq1 antibody. Data are representative of at least three independent experiments. (D) Co-immunoprecipitation of Sis1 and Rnq1. Sis1 was immunoprecipitated from cell lysates using an αSis1 antibody (gift from E. Craig), analyzed by SDS-PAGE, and immunoblotted with αRnq1 and αSis1 antibodies. The input of the total cell lysate (I) represents 10% of the sample that was bound by the αSis1 antibody (B). The bound fraction from the same sample not incubated with antibody (N) was used as a control.

[*PSI*+] variants are easily distinguished by a set of parameters that define an equilibrium between fiber growth and fiber fragmentation [20]. We asked whether these parameters could similarly distinguish a set of previously published [*RNQ*+] variants. This set consisted of four s.d. [*RNQ*+] variants having [*PSI*+] induction levels from low to very high, and one m.d. [*RNQ*+] variant with a high level of [*PSI*+] induction [44]. We first examined whether differences in the fragmentation of the [*RNQ*+] variants could explain the differences in [*PSI*+] inducibility. The properties that dictate fragmentation often include aggregate stability, aggregate size, and chaperone interactions. To test the stability of the Rnq1 aggregates, we subjected [*RNQ*+] [*psi*−] cell lysates to a gradient of increasing temperature, as different amyloid conformations can have different melting temperatures (T_m_) [19]. In agreement with previous studies [33], [49], [51], we found that only the m.d. high [*RNQ*+] variant was thermal labile, having a much lower T_m_ (∼58°C) as compared to the similar T_m_ (∼86°C) of all the s.d. [*RNQ*+] variants (Figure 1A). We also analyzed the sensitivity of Rnq1 aggregates to protease digestion, as work with the mammalian PrP^Sc^ prion has shown that prion strains can display different sensitivities to digestion with proteinase K (PK) [9]. Strains of α-synuclein also show different levels of PK sensitivity [10]. After incubating cell lysates with PK at 37°C, we again found that only m.d. high [*RNQ*+] was an outlier in displaying increased PK resistance as compared to the s.d. [*RNQ*+] variants (Figure 1B and Figure S1).

We then analyzed the average size distribution of the Rnq1 aggregates using semi-denaturing agarose gel electrophoresis (SDD-AGE), which resolves the Rnq1 aggregated species. By electrophoretically separating the protein longer, we observed some more distinctions between the [*RNQ*+] variants than previously described [51]. There was a gradual decrease in aggregate size between s.d. low, s.d. medium, and s.d. high [*RNQ*+] (notice the aggregates of s.d. low [*RNQ*+] that appear stuck in the well) (Figure 1C). However, both s.d. very high and m.d. high [*RNQ*+] had predominantly larger aggregates.

Another major factor that distinguishes the ability of yeast prion variants to fragment *in vivo* is their interaction with chaperones. Prion replication requires recognition of prion aggregates by the Hsp40 co-chaperone Sis1 and fragmentation by Hsp104 to create “seeds” (or propagons) that cause further monomeric conversion to the prion state [12], [53]. Sis1 interacts with Rnq1 specifically in [*RNQ*+] cells [54] and this interaction is required for propagation of [*RNQ*+] [55]. Additionally, more Sis1 is associated with Rnq1 aggregates as compared to Sup35 aggregates [56], which may suggest that Sis1 plays a more significant role in the propagation of [*RNQ*+] as compared to [*PSI*+]. Therefore, as another measure of fragmentability, we asked whether the level of Sis1 associated with Rnq1 differed between [*RNQ*+] variants. To test this, we immunoprecipitated Sis1 from [*rnq*−] cells or cells propagating the [*RNQ*+] variants and immunoblotted for Rnq1. While Sis1 did not co-immunoprecipitate Rnq1 in [*rnq*−] cells, Sis1 and Rnq1 were similarly associated in all [*RNQ*+] cells (Figure 1D), in agreement with a previous study [52]. Furthermore, all the [*RNQ*+] variants depended on Sis1 expression to similar levels for their maintenance (data not shown).

These data do not preclude the possibility that the [*RNQ*+] variants are fragmented to different extents. However, they show that the standard parameters used to monitor fragmentation neither distinguish the [*RNQ*+] variants, as they do for [*PSI*+] variants, nor correlate to [*PSI*+] inducibility.

### Fiber growth parameters do not explain phenotypic differences of [*RNQ*+] variants

In addition to differences in fiber fragmentation, [*PSI*+] variants also show differences in fiber growth parameters. Knowing the functional role of Sup35 in translation termination allows these differences to be easily monitored phenotypically. The most common means involves colorimetrically measuring the degree of nonsense suppression of the *ade1-14* allele that has a premature stop codon in the *ADE1* gene [57]. The large soluble pool of Sup35 in [*psi*−] cells allows for faithful translation termination at the premature stop codon. This results in the accumulation of a metabolic intermediate and incomplete synthesis of adenine, thereby making these cells unable to grow on medium lacking adenine, and colonies grown on rich media are red. By contrast, Sup35 aggregates in strong [*PSI*+] cells readily recruit and sequester soluble Sup35 into aggregates to cause nonsense suppression [15], [58]. Hence, strong [*PSI*+] cells have less soluble Sup35, propagate a mitotically stable aggregate structure, and form light pink or white colonies that grow well on medium lacking adenine. Weak [*PSI*+] cells, on the other hand, have an intermediate phenotype. Thus, the fiber growth characteristics of [*PSI*+] variants are often monitored by: mitotic stability, the amount of soluble protein, and the resulting [*PSI*+] phenotype.

**Figure 2:**
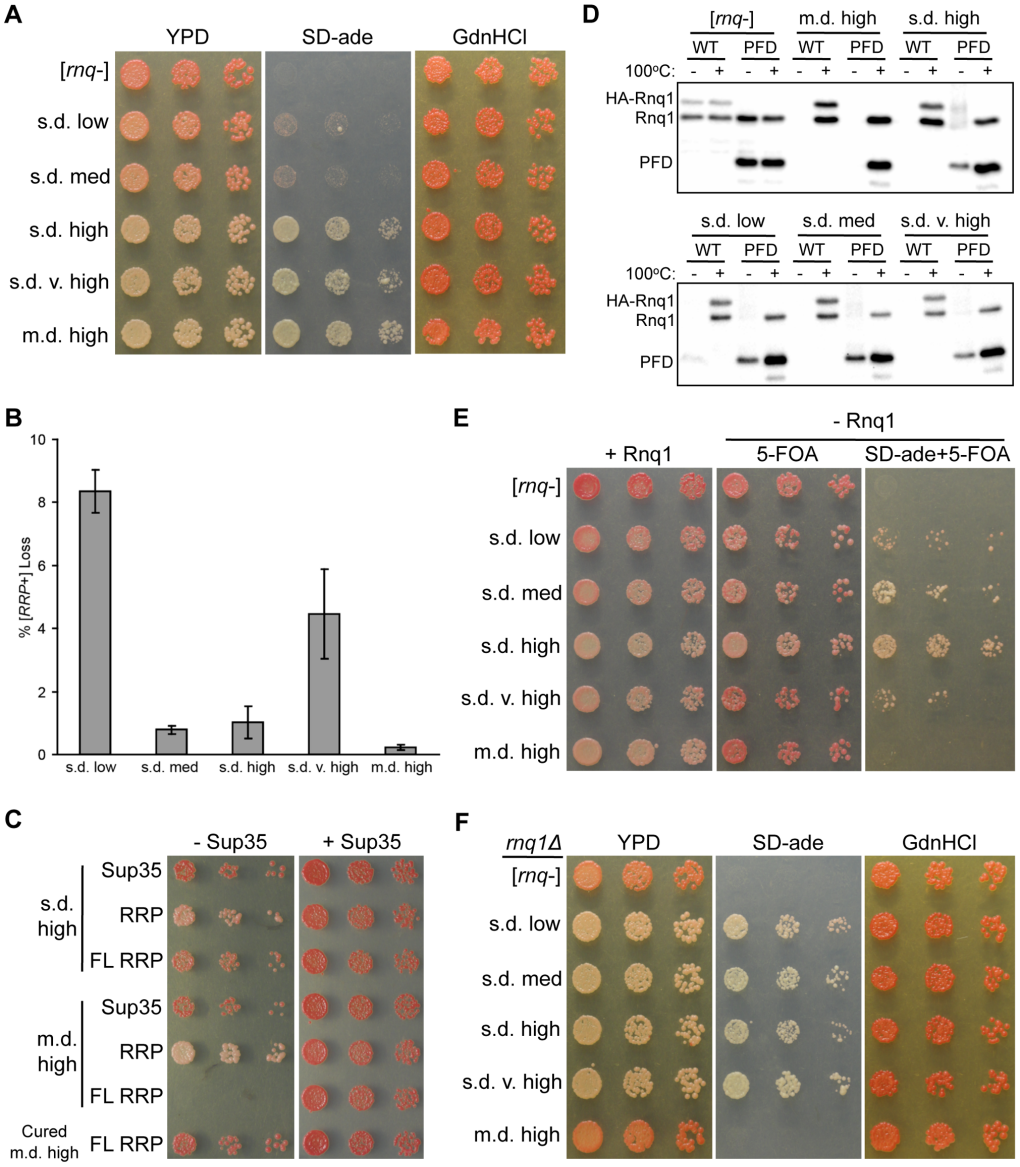
The non-prion forming domain of Rnq1 shows variant-specific influence on fiber growth. (A) Higher [*RNQ*+] variants confer stronger [*RRP*+] phenotypes. Cells harboring RRP and the indicated [*RNQ*+] variant were normalized by OD_600_, serially diluted five-fold, and spotted onto YPD, SD-ade, and YPD+3 mM GdnHCl (GdnHCl). Representative spottings are shown. (B) Mitotic stability of [*RNQ*+] variants. Averages indicate the percentage of colonies that lost [*RRP*+] and error bars represent standard error of the mean calculated from at least three independent experiments. (C) Expression of full-length (FL) RRP in m.d. high [*RNQ*+] cells is lethal. Normalized numbers of yeast cells propagating s.d. high [*RNQ*+], m.d. high [*RNQ*+], or cured of m.d. high [*RNQ*+], and expressing Sup35, RRP, or FL RRP were serially diluted five-fold and spotted to select for loss (− Sup35) or co-expression (+ Sup35) of wild-type Sup35. Representative spottings are shown. (D) Rnq1-PFD readily joins pre-existing Rnq1 aggregates of m.d. high [*RNQ*+] as compared to s.d. [*RNQ*+] variants. Cells transformed with plasmids expressing *HA-RNQ1* or *RNQ1-PFD* from the *GAL1* promoter were subcultured in galactose media. Lysates were incubated for five minutes at 100°C (+) or at room temperature (−), followed by SDS-PAGE and western blot using an αRnq1 antibody. Data are representative of three independent experiments. (E) RRP is not sufficient to propagate m.d. high [*RNQ*+] without expression of Rnq1. Normalized numbers of *rnq1Δ* cells harboring RRP were serially diluted five-fold and spotted to select for maintenance of Rnq1 expression (+ Rnq1), or loss of Rnq1 (− Rnq1) on media to monitor color (5-FOA) and nonsense suppression (SD-ade+5-FOA) phenotypes. Representative spottings are shown. (F) Loss of Rnq1 results in altered [*RRP*+] phenotypes. Normalized numbers of *rnq1Δ* cells harboring RRP that lost Rnq1 expression, as shown in (E), were serially diluted five-fold and spotted onto YPD, SD-ade, and GdnHCl media to monitor the stable [*RRP*+] phenotypes. Representative spottings are shown.

In contrast to Sup35, the functional role of Rnq1 is not known. Hence, in order to phenotypically monitor [*RNQ*+], we previously developed a chimeric protein called the [*RNQ*+] Reporter Protein (RRP) [49], which consists of the Rnq1-PFD(153-405) fused to the middle and C-terminal domains of Sup35 that provide the GTPase activity required for translation termination [59]. RRP allows us to monitor the [*RNQ*+] prion state by colony color phenotype in a manner analogous to [*PSI*+]. RRP is functional in translation termination in [*rnq*−] cells that remain red, while it co-aggregates with Rnq1 in [*RNQ*+] cells to generate colonies that are phenotypically pink or white [33], [34]. Thus, the [*RRP*+] phenotype, like the [*PSI*+] phenotype, serves as a measure of the prion variant-specific sequestration of monomeric protein. Hence, to examine fiber growth parameters, we first determined the [*RRP*+] phenotype for the set of [*RNQ*+] variants studied in this work. Interestingly, we found that the three “higher” [*RNQ*+] variants had a stronger nonsense suppression phenotype as compared to s.d. low and s.d. medium [*RNQ*+] (Figure 2A). Previous work demonstrated that s.d. very high [*RNQ*+] cells had the greatest amount of soluble Rnq1 [44]. Hence, this suggests that, in contrast to other published [*RNQ*+] variants [34], the [*RRP*+] phenotype of the set of [*RNQ*+] variants in this study does not correlate to the level of soluble Rnq1. Moreover, it was surprising that the [*RRP*+] phenotype correlated to [*PSI*+] inducibility for these [*RNQ*+] variants, as this was not the case for other [*RNQ*+] variants [33], [34], or for several other prion properties of [*RNQ*+] (this work and [44], [51], [52]).

The mitotic stability of prion variants is another measure of fiber growth as this property relies on the degree to which monomer is sequestered into aggregates. This property has been shown to distinguish [*PSI*+] variants and other [*RNQ*+] variants [15], [33], [34]. Hence, to test the mitotic stability of the [*RNQ*+] variants, we plated overnight cultures of cells expressing RRP and propagating each of the [*RNQ*+] variants onto rich media, and then counted the number of colonies that were red or had red sectors (indicating loss of the [*RRP*+] phenotype and the [*RNQ*+] prion). We found that both s.d. low and s.d. very high [*RNQ*+] showed higher levels of mitotic loss compared to the other three [*RNQ*+] variants (Figure 2B). This loosely correlates with the amount of soluble Rnq1 present in these cells: s.d. low and s.d. very high [*RNQ*+] have been shown to have the greatest amount of soluble Rnq1 [44]. However, because s.d. low [*RNQ*+] has less soluble protein as compared to s.d. very high [*RNQ*+] [44], we would have expected s.d. low [*RNQ*+] to have higher mitotic stability. Hence, mitotic stability did not correlate to either the [*RRP*+] phenotypes or the level of soluble Rnq1 of this set of [*RNQ*+] variants, in contrast to what was shown with [*PSI*+] variants and other [*RNQ*+] variants [34], [58].

### The region outside of the Rnq1-PFD has variant-specific influence on fiber growth

While regions outside of PFDs remain under-studied, various work has implicated non-PFD regions as affecting prion propagation [28], [60]. Indeed, the N-terminal domain that lies outside of the putative Rnq1-PFD can influence the propagation of [*RNQ*+] [49], [61], [62]. As such, to gain insight into how the [*RNQ*+] variants are physically distinct, we hypothesized that the Rnq1 N-terminal domain might impact [*RNQ*+] propagation in a variant-specific manner. To address this, we used a modified version of RRP containing full-length (FL) Rnq1 (aa 1-405) fused to the MC domains of Sup35, in contrast to the original version of RRP that contained just the Rnq1-PFD (aa 153-405). Using *sup35Δ* cells that were complemented by episomal *SUP35*, we used a plasmid shuffle technique to replace Sup35 with plasmids expressing RRP, FL RRP, or Sup35. Strikingly, FL RRP was not able to functionally replace Sup35 in cells propagating the m.d. high [*RNQ*+] variant (Figure 2C). Importantly, there was no effect on viability when cells still expressed Sup35. Moreover, the inviability depended on the presence of both the m.d. high [*RNQ*+] variant and the Rnq1 protein, as eliminating (curing) the prion or deleting *RNQ1* before replacing Sup35 with FL RRP resulted in viable cells (Figures 2C and S2). (As expected, the cells were white in the latter case, indicating that FL RRP propagates the aggregated structure of m.d. high [*RNQ*+] in the absence of the Rnq1 protein since FL Rnq1 is still expressed as part of FL RRP.) In light of previous work showing that m.d. high [*RNQ*+] cells have relatively little soluble Rnq1 [44], these data suggest that the presence of the Rnq1 N-terminus on FL RRP promotes sequestration of FL RRP into Rnq1 aggregates to such a large extent that there is too little soluble FL RRP to function in translation termination. In contrast, [*rnq*−] cells and cells propagating each of the s.d. [*RNQ*+] variants were viable when expressing FL RRP (Figures 2C and S3). In fact, cells propagating each of the s.d. [*RNQ*+] variants and harboring FL RRP were darker pink as compared to the equivalent cells expressing RRP, thus indicating that translation termination was more efficient.

Based on the different phenotypes that resulted from using RRP versus FL RRP, we wanted to more directly test, without the use of RRP, whether the Rnq1 N-terminus influenced the ability of newly synthesized Rnq1 to join pre-existing Rnq1 aggregates. We used a galactose-inducible promoter to express HA-tagged Rnq1 or Rnq1-PFD in cells that are grown in media containing galactose. Then we monitored the ability of Rnq1 monomer to join untagged pre-existing aggregates of the different [*RNQ*+] variants using a well-trap assay. This assay allows us to easily determine the amount of soluble Rnq1 in the cell, as aggregated Rnq1 is retained in the wells of an SDS-PAGE gel when samples are not boiled [63]. As expected for [*rnq*−] cells, upon inducing expression of HA-Rnq1 or Rnq1-PFD, we found that all pre-existing and newly synthesized Rnq1 protein was soluble (Figure 2D). By contrast, all newly synthesized HA-Rnq1 joined the pre-existing aggregates for each of the [*RNQ*+] variants, as indicated by the absence of a band in the unboiled lanes. Interestingly, we saw variant-specific effects when we expressed just the Rnq1-PFD. For m.d. high [*RNQ*+], newly synthesized Rnq1-PFD, like HA-Rnq1, completely joined the pre-existing aggregates in the given time duration. All of the s.d. [*RNQ*+] variants, on the other hand, maintained a soluble pool of Rnq1-PFD. These data indicate that recruitment of Rnq1-PFD monomers into aggregates occurs more readily for m.d. high [*RNQ*+] in comparison to the s.d. [*RNQ*+] variants. Additionally, it suggests that the Rnq1 N-terminus is important for facilitating monomer addition and fiber growth with the s.d. [*RNQ*+] variants, similar to what we demonstrated above for m.d. high [*RNQ*+] using FL RRP.

Finally, we wanted to test whether this influence of the Rnq1 N-terminal domain on fiber growth was necessary for propagation of the [*RNQ*+] variants. To do this, we deleted *RNQ1* in RRP-expressing yeast cells that were complemented with a *URA3*-marked plasmid expressing *RNQ1*. Cells that lose this plasmid would express only the Rnq1-PFD as part of RRP, and would not express the Rnq1 N-terminal domain. After growing cultures in non-selective media, we plated [*rnq*−] cells and cells propagating each of the [*RNQ*+] variants on media to select for either maintenance (SD-ura) or loss (5-FOA) of the *RNQ1* plasmid. In agreement with our previous results [49], m.d. high [*RNQ*+] did not propagate when Rnq1 was lost, as it was phenotypically [*rnq*−] on 5-FOA medium and did not grow on medium lacking adenine and containing 5-FOA (Figure 2E). This suggests that the Rnq1-PFD is not sufficient to propagate this variant. In stark contrast, all of the s.d. [*RNQ*+] variants could still maintain [*RNQ*+], albeit to varying degrees. This indicates that propagation of m.d. high [*RNQ*+] has a greater reliance on the non-prion-forming N-terminal domain than the s.d. [*RNQ*+] variants. Furthermore, it suggests that propagation of the s.d. [*RNQ*+] variants might differentially depend on the presence of the N-terminal domain. Indeed, we passaged the *rnq1Δ* cells harboring the s.d. [*RNQ*+] variants that had lost the *RNQ1* plasmid and found that, after further growth, these cells did not maintain the same [*RRP*+] phenotype as the *RNQ1* [*RNQ*+] cells expressing RRP (compare Figure 2A to Figure 2F). For instance, both s.d. low and s.d. medium [*RNQ*+] had a much stronger nonsense suppression phenotype as compared to their phenotype when Rnq1 was present.

Taken together, these data suggest that the [*RNQ*+] variants show differences in fiber growth parameters, which are mediated, in part, by the Rnq1 N-terminal domain. However, despite these differences in properties of fiber growth and fragmentation, these data do not wholly distinguish the [*RNQ*+] variants or explain their differential ability to induce [*PSI*+]. This is in line with previous work that also shows a lack of correlation [52]. Hence, these data highlight the complex nature of both [*PSI*+] induction and the phenotypic consequences that are possible with distinct aggregate structures.

### [*RNQ*+] variants show differential reliance on amyloidogenic regions

**Figure 3:**
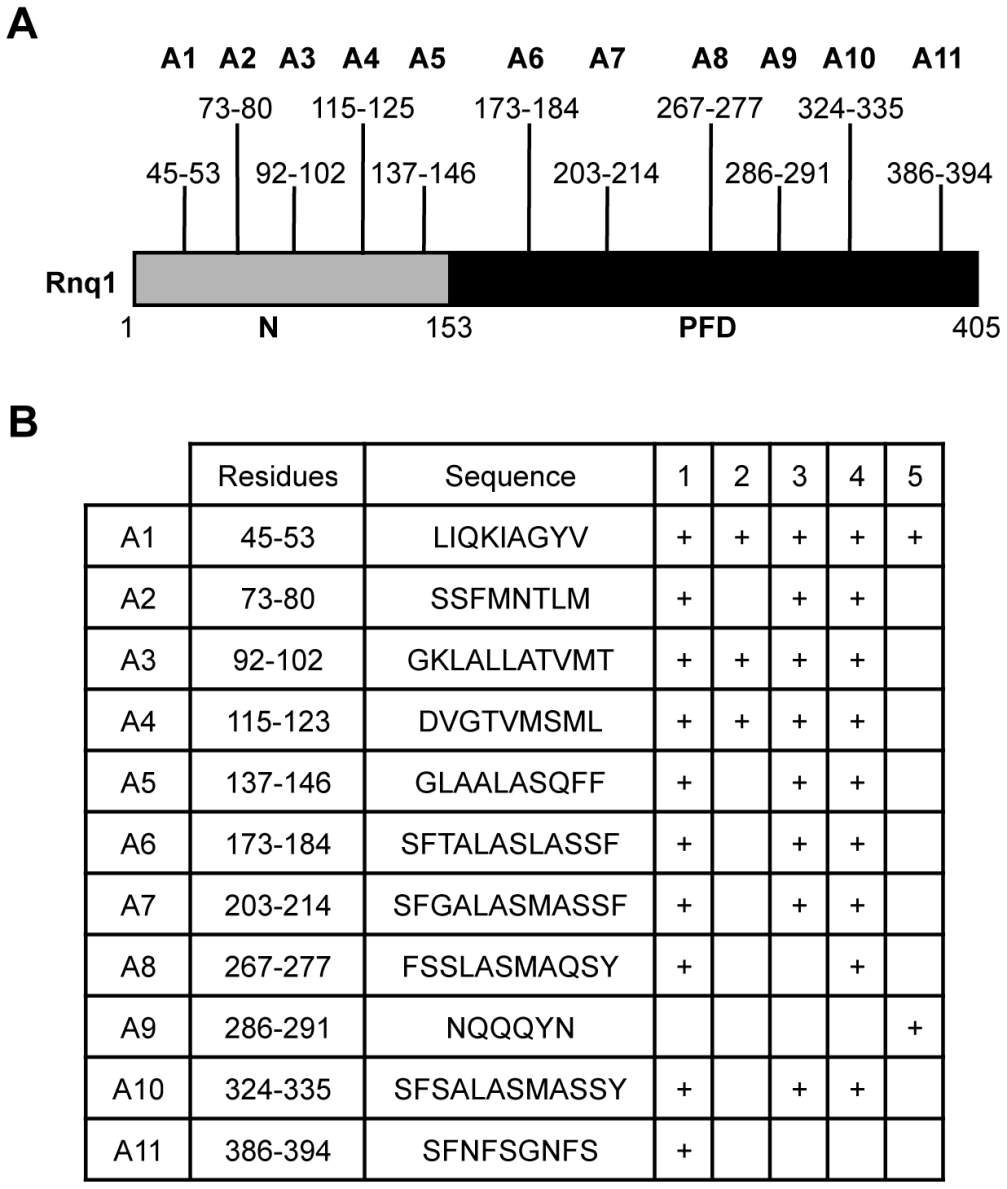
Consensus amyloidogenic regions of Rnq1 identified by prediction algorithms. (A) Diagram of the Rnq1 protein highlighting the N-terminal domain (N) between residues 1-152, the putative prion-forming domain (PFD) between 153-405, and the identified amyloidogenic regions. (B) Residues and sequences of the predicted amyloidogenic regions and which algorithm identified that region: 1) Zyggregator (http://www-vendruscolo.ch.cam.ac.uk/zyggregator.php); 2) PASTA (http://biocomp.bio.unipd.it/pasta/); 3) TANGO (http://tango.crg.es/); 4) Aggrescan (http://bioinf.uab.es/aggrescan/); 5) WALTZ (http://waltz.switchlab.org/).

To help elucidate the basis of the structural diversity of the [*RNQ*+] variants and the phenotypic differences they modulate, we postulated that each of the [*RNQ*+] variants, like [*PSI*+], has a different part of the primary structure that is important for prion propagation. Propagation of strong [*PSI*+] incorporates fewer residues of the Sup35-PFD in the amyloid core as compared to weak [*PSI*+], and this difference is tightly correlated to the biochemical and phenotypic differences seen between [*PSI*+] variants [21], [22], [28]. Hence, determining the regions of Rnq1 that are important for propagation of each of the [*RNQ*+] variants would provide insight into how the [*RNQ*+] variants are structurally different and can mediate differences in [*PSI*+] formation. To help identify these sequence elements, we used five different algorithms that were developed to find regions of a protein predicted to facilitate the formation of amyloid [64]–[68]. These algorithms found 11 putative amyloidogenic regions spread throughout both the N-terminal domain and the PFD of Rnq1 (Figure 3). We will refer to these regions hereafter as A1 through A11.

We set out to analyze the influence of these regions on the propagation of the different [*RNQ*+] variants. We mutated each of these regions in its entirety to alanine to make 11 Rnq1 mutant constructs and confirmed expression levels similar to wild-type (WT) Rnq1 (Figure S4). After using a plasmid shuffle technique to replace WT Rnq1 with these mutants in cells propagating each of the [*RNQ*+] variants, we then examined three biochemical properties commonly used to monitor prion replication: 1) the relative distribution of Rnq1 aggregates using SDD-AGE, 2) the amount of soluble Rnq1 by solubility assay, and 3) the thermal stability of Rnq1 aggregates. These assays provide complementary information about the propagation of [*RNQ*+]; for example, detection of monomeric Rnq1 with SDD-AGE is variable for unknown reasons, thus using the solubility assay is informative. Additionally, since these assays examine different aspects of prion propagation, we hypothesized that we might see a difference by one assay and not another. Therefore, collectively, these assays allowed us to make conclusions about how mutation of the amyloidogenic regions in Rnq1 affected [*RNQ*+] propagation.

**Figure 4:**
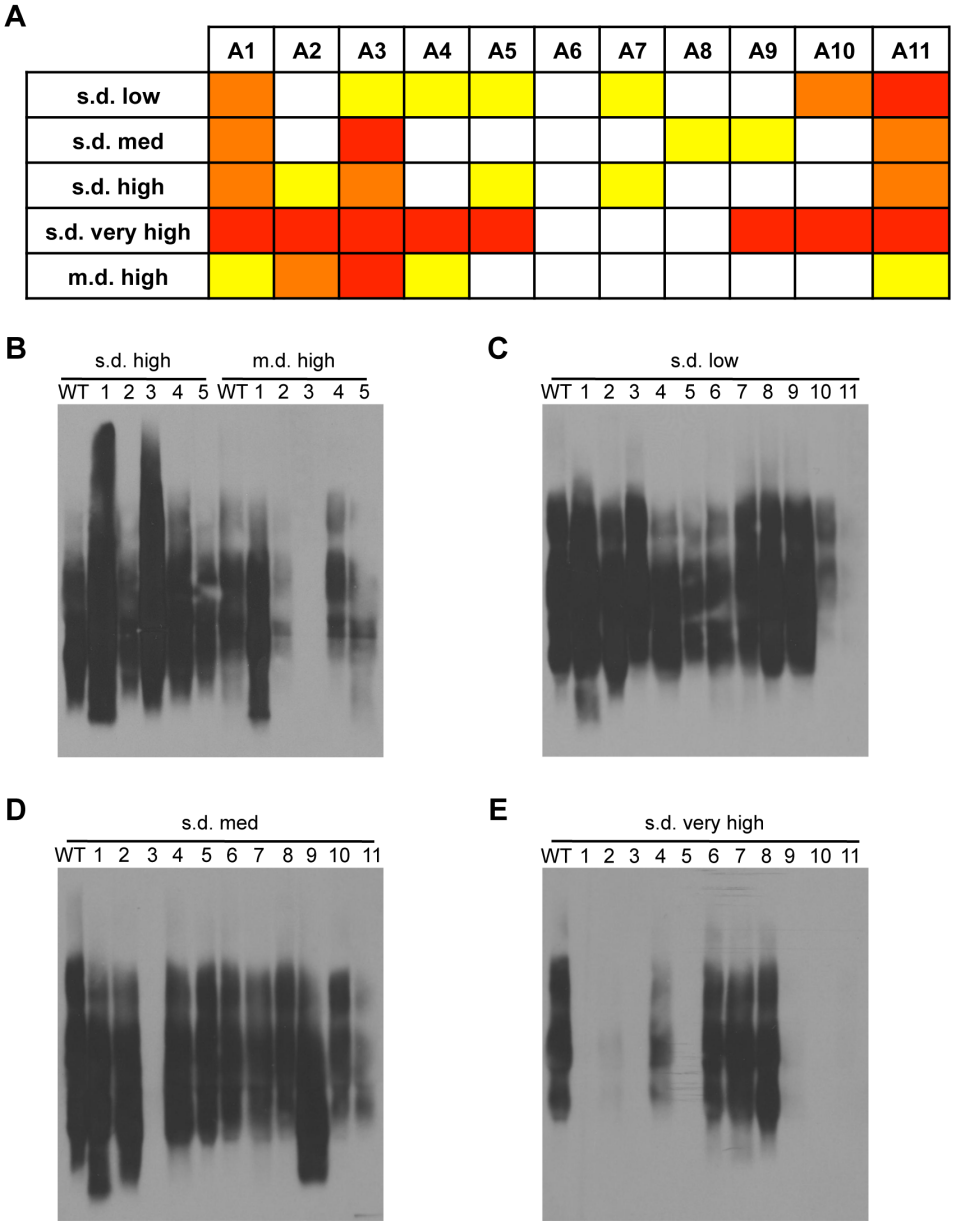
Distinct regions of Rnq1 are important for each [*RNQ*+] variant. (A) Summary of how disruption of the amyloidogenic regions by alanine mutations affects propagation of the [*RNQ*+] variants. Mutants are categorized as having no effect (white), mild effect (yellow), moderate effect (orange), or not propagating [*RNQ*+] (red) after successive passaging. These effects on [*RNQ*+] propagation were summarized from at least three independent experiments of each of the following assays: solubility assay, thermal stability, and SDD-AGE. See Figure S5 for more detail. If only one assay showed mild effects, e.g. SDD-AGE of s.d. low [*RNQ*+] cells harboring the A6 mutant, this was scored as having no effect in this summary table. (B) Mutation of the Rnq1 N-terminal domain shows [*RNQ*+] variant-dependent effects. Lysates from cells propagating s.d. high or m.d. high [*RNQ*+] and expressing wild-type (WT) Rnq1 or the N-terminal alanine mutants (A1–A5) were resolved by SDD-AGE, followed by western blot using an αRnq1 antibody. (C–E) Mutation of Rnq1 amyloidogenic regions differentially affects [*RNQ*+] variants. Yeast lysates propagating (C) s.d. low [*RNQ*+], (D) s.d. medium [*RNQ*+], or (E) s.d. very high [*RNQ*+] expressing WT Rnq1 or the set of alanine mutants (A1–A11) were analyzed by SDD-AGE and western blot with an αRnq1 antibody.

For each assay, we categorized the reproducible effects of the alanine mutants as mild, moderate, curing [*RNQ*+], or having no effect (Figure S5). Taking all three assays into account, we then scored the overall influence of disrupting the amyloidogenic regions on each [*RNQ*+] variant (Figure 4A). As expected, since our data above showed the involvement of the N-terminus in [*RNQ*+] propagation, we found that the N-terminal alanine mutants revealed regions of both common and unique importance for the [*RNQ*+] variants. For instance, mutation of the A1 or A3 regions both affected the biochemical properties of all of the [*RNQ*+] variants. However, these mutants also showed variant-specific effects. With s.d. high [*RNQ*+], for example, expression of the A1 and A3 mutants resulted in a dramatic shift in relative aggregate size distribution, but the other N-terminal mutations had minimal effect on propagation of s.d. high [*RNQ*+] (Figure 4B). By contrast, the A3 mutant cured the m.d. high [*RNQ*+] variant, while the A1 mutant resulted in a shift in aggregate size. These data show that particular regions within the non-PFD N-domain have differential roles on prion propagation depending on what [*RNQ*+] variant is present. This indicates that this domain helps define the physical basis of distinct [*RNQ*+] variants.

Prion-forming domains are historically considered the region that forms the amyloid core of prion aggregates [50], [69]. As such, PFDs are predicted to encode variant-specific differences, as is the case for weak and strong [*PSI*+] [21], [22], [28]. Indeed, we have previously shown that deletion of part of the Rnq1-PFD differentially affects the propagation of some [*RNQ*+] variants [49]. Hence, we expected to uncover conformation-specific effects with mutation of the amyloidogenic regions in the Rnq1-PFD. We found that mutation of the A11 region affected propagation of all of the [*RNQ*+] variants, but to varying degrees. For instance, the A11 mutant was the only mutant that cured s.d. low [*RNQ*+] (Figure 4C). On the other hand, none of the PFD alanine mutants cured s.d. medium [*RNQ*+], only mutation of the N-terminal A3 region did (Figure 4D). This suggests that the A3 and A11 regions are the most important of the amyloidogenic regions for propagation of s.d. medium and s.d. low [*RNQ*+], respectively. Strikingly, disruption of 8 of the 11 amyloidogenic regions affected propagation of s.d. very high [*RNQ*+], with several alanine mutants quickly curing this variant (A1, A5, A10, A11) (Figure 4E). Additionally, upon further growth, the s.d. very high [*RNQ*+] variant was also cured by mutation of the A2, A3, A4, and A9 regions. Importantly, loss of [*RNQ*+] was confirmed by the absence of SDS-resistant aggregates by SDD-AGE after re-transforming a plasmid expressing WT Rnq1 and selecting for loss of the Rnq1 alanine mutant plasmid (Figure S6).

Overall, it has previously been shown that different, non-adjacent Q/N-rich regions of the Rnq1 protein make differential contributions to [*RNQ*+] propagation [70]. Our data now show that a unique set of non-contiguous regions, even regions that are not Q/N-rich, defines the propagation of the [*RNQ*+] variants. This strongly supports the hypothesis that the [*RNQ*+] variants propagate distinct structural conformations and likely mediate differential induction of [*PSI*+] by exposing a different structural template.

### [*RNQ*+] variants have additional Sis1 binding sites

**Figure 5:**
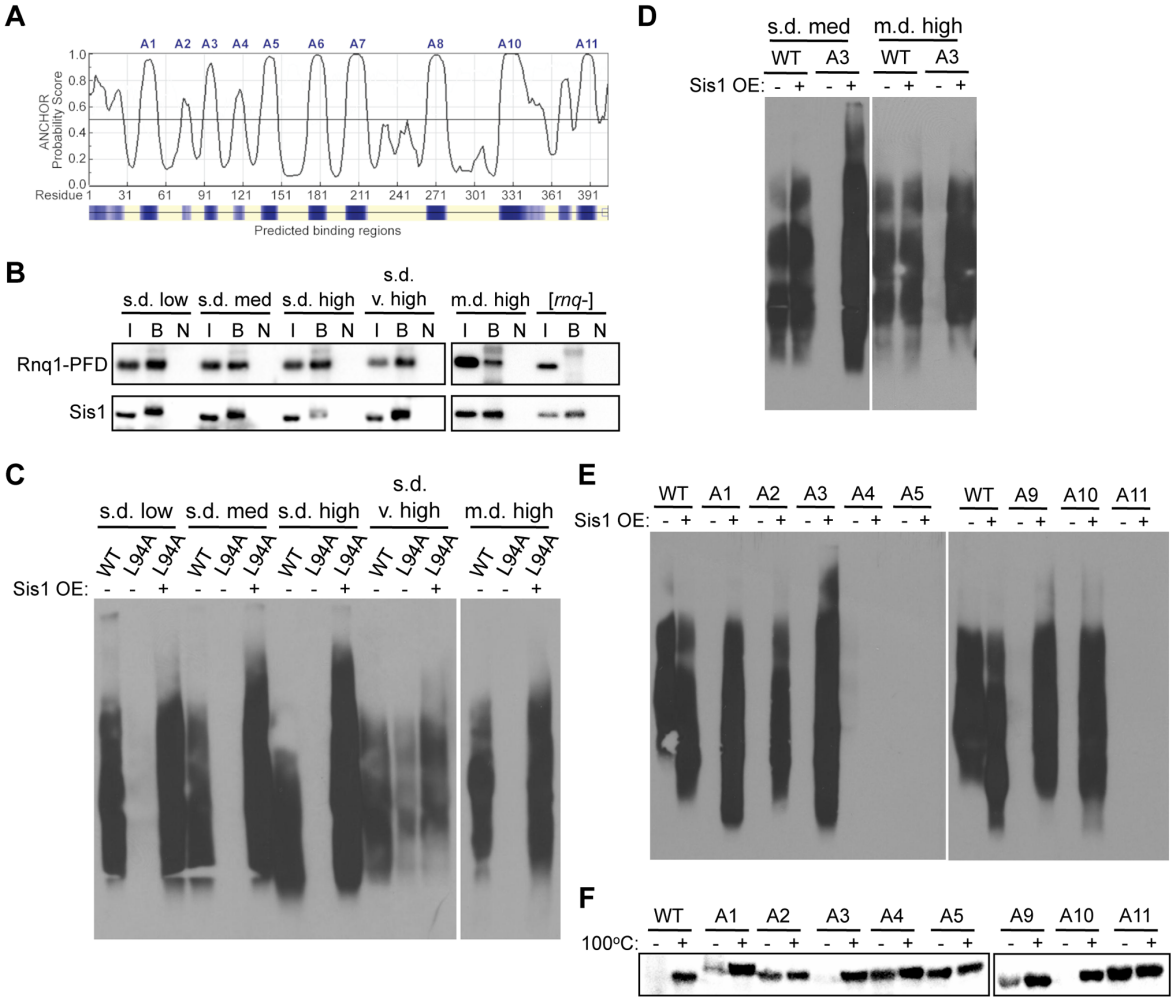
Rnq1 amyloidogenic regions differentially modulate interactions with Sis1. (A) Prediction of protein interaction sites in Rnq1 using the ANCHOR algorithm (http://anchor.enzim.hu/). Denoted at the top are the amyloidogenic regions A1–A11. (B) Co-immunoprecipitation of Sis1 and Rnq1(132-405). Sis1 was immunoprecipitated using an αSis1 antibody (gift from E. Craig) from cell lysates expressing Rnq1(132-405) in place of WT Rnq1, followed by SDS-PAGE and immunoblotting with αRnq1 and αSis1 antibodies. The input of the total cell lysate (I) represents 10% of the sample that was bound by the αSis1 antibody (B). The bound fraction from the same sample not incubated with antibody (N) was used as a control. (C) Rnq1-L94A differentially affects [*RNQ*+] variant propagation and is rescued by Sis1 over-expression. Cell lysates with (+) or without (−) over-expression (OE) of Sis1 and propagating the indicated [*RNQ*+] variant with WT Rnq1 or Rnq1-L94A were subjected to SDD-AGE and western blot using an αRnq1 antibody. (D–F) Elimination of [*RNQ*+] by mutation of Rnq1 amyloidogenic regions is differentially rescued by Sis1 over-expression. As in (C) for (D) cells propagating s.d. medium [*RNQ*+] or m.d. high [*RNQ*+] and expressing WT Rnq1 or the A3 mutant, or (E) cells propagating s.d. very high [*RNQ*+] expressing WT Rnq1 or mutants A1–A5 or A9–A11. (F) Yeast lysates propagating s.d. very high [*RNQ*+], over-expressing Sis1, and expressing WT Rnq1, or mutants A1–A5 or A9–A11 were incubated for five minutes at 100°C (+) or at room temperature (−), followed by SDS-PAGE and western blot using an αRnq1 antibody. Data are representative of at least three independent experiments.

We wanted to better understand the role that these putative amyloidogenic regions play in propagating the different conformations of [*RNQ*+]. We noted that most of these regions were primarily hydrophobic, with some regions being previously identified as separating the Q/N-rich regions of the Rnq1-PFD [70]. It was unsurprising that the prediction algorithms identified these regions, as hydrophobic residues are generally recognized as sites that facilitate protein interactions, whether normal or abnormal interactions [71]. We conjectured two predominant ways that mutation of these amyloidogenic regions could affect the prion replication cycle of [*RNQ*+]: 1) disrupting the aggregate structure, or 2) disrupting the ability of chaperones to bind the Rnq1 aggregates. Interestingly, one of the amyloidogenic regions of Rnq1, A3 (aa 92-102), was previously identified as containing a binding site for the Hsp40 chaperone Sis1 [72]. Disruption of this site using the Rnq1-L94A mutation impairs Sis1 binding and eliminates the [*RNQ*+] prion [72], [73]. Moreover, the bacterial Hsp40 has been predicted to bind several different hydrophobic stretches in each protein of nearly the whole proteome [74], [75]. In line with this, we found that the ANCHOR algorithm, which predicts protein-binding sites in disordered stretches [76], [77], identified all of our amyloidogenic regions (except the lone hydrophilic A9 region) to be putative chaperone-binding sites (Figure 5A). As such, we first wanted to test whether Sis1 could bind other regions of Rnq1 outside of the known A3 binding site. To do this, we expressed Rnq1-PFD (which lacks the A3 binding site) in place of full-length Rnq1 in cells propagating each of the [*RNQ*+] variants. Upon immunoprecipitating Sis1, we found that Sis1 could still bind the PFD of Rnq1 in [*RNQ*+] cells, but not in [*rnq*−] cells (Figure 5B), suggesting that some of the amyloidogenic regions in the Rnq1-PFD may serve as additional binding sites for Sis1. Interestingly, in contrast to Figure 1D in which Sis1 bound equal levels of full-length Rnq1 in all of the [*RNQ*+] variants, there was distinctly less Rnq1-PFD bound to Sis1 in the m.d. high [*RNQ*+] variant. While we confirmed that these cells were still [*RNQ*+] at this time point (data not shown), this suggests that propagation of the m.d. high [*RNQ*+] conformer might uniquely require the presence of the A3 binding site. Indeed, this would provide some mechanistic explanation for our finding that the Rnq1-PFD as part of RRP is not sufficient for propagation of the m.d. high [*RNQ*+] variant in particular (Figure 2E).

### Rnq1 amyloidogenic regions differentially contribute to the interaction of Sis1 with the [*RNQ*+] variants

Since Sis1 only binds Rnq1 in [*RNQ*+] cells, it is challenging to distinguish whether loss of Sis1 binding results in curing [*RNQ*+], or if curing [*RNQ*+] by disrupting some other aspect required for prion propagation results in loss of Sis1 binding. As the A3 region was previously shown to be a major binding site for Sis1, we hypothesized that disruption of this region by the Rnq1-L94A mutation would provide a useful comparison for examining how mutation of the amyloidogenic regions influences Sis1 binding and propagation of [*RNQ*+]. Therefore, to determine the effect of Rnq1-L94A on [*RNQ*+] propagation, we expressed Rnq1-L94A in place of WT Rnq1 in cells propagating each of the [*RNQ*+] variants and monitored the maintenance of [*RNQ*+] by SDD-AGE. Like the variant-specific differences we observed above, we found that Rnq1-L94A also differentially affected the [*RNQ*+] variants: s.d. very high [*RNQ*+] could still propagate while the other four [*RNQ*+] variants were cured (Figure 5C). However, upon growing cells additional generations, we found that s.d. very high [*RNQ*+] was eventually cured as well (data not shown). This delayed curing suggests that s.d. very high [*RNQ*+] does not rely on the L94 Sis1 binding site in the A3 region to the same extent as the other [*RNQ*+] variants. Importantly, over-expressing Sis1 before replacing WT Rnq1 with Rnq1-L94A rescued propagation of the [*RNQ*+] variants, as an SDS-resistant species was still maintained (Figure 5C) and was transmissible (data not shown). Therefore, these data indicate that over-expression of Sis1 compensates for the disruption of Sis1 binding caused by the Rnq1-L94A mutation.

In using Rnq1-L94A as a model of how disruption of Sis1 binding affects [*RNQ*+], we hypothesized that if mutation of the Rnq1 amyloidogenic regions impaired the binding of Sis1, then Sis1 over-expression would at least partially rescue the propagation of the [*RNQ*+] variants. As a corollary, we postulated that if the mutations instead disrupted the Rnq1 aggregate structure, then these mutants would still cure [*RNQ*+] despite Sis1 being over-expressed. Therefore, as we did using Rnq1-L94A, we over-expressed Sis1 before replacing WT Rnq1 with the alanine mutants and tested whether propagation of the [*RNQ*+] variants was rescued using SDD-AGE to monitor the presence of SDS-resistant aggregates. As expected from our data with Rnq1-L94A, Sis1 over-expression rescued the propagation of m.d. high [*RNQ*+] and s.d. medium [*RNQ*+] that were both cured by mutation of the A3 region, which encompasses L94 (Figure 5D).

Propagation of s.d. very high [*RNQ*+], on the other hand, was impaired by mutation of several different amyloidogenic regions (Figure 4). This allowed us to gain a more complete picture of how multiple regions of Rnq1 influence propagation of this prion variant. Strikingly, Sis1 over-expression differentially rescued propagation with these mutants, even when comparing N-terminal and C-terminal mutants (Figure 5E). Sis1 over-expression did not rescue s.d. very high [*RNQ*+] propagation when the A4, A5, or A11 regions were mutated to alanine. This suggests that these regions likely disrupt the aggregate structure of s.d. very high [*RNQ*+], thereby impairing the recruitment and conversion of monomeric Rnq1. By contrast, Sis1 over-expression did rescue the propagation of s.d. very high [*RNQ*+] when the N-terminal A1, A2, and A3 regions were mutated, along with the C-terminal A9 and A10 regions. Thus, these data support our hypothesis that mutation of these regions impairs Sis1 binding, which is overcome by Sis1 over-expression.

In order to more finely monitor the degree to which Sis1 over-expression rescued propagation of s.d. very high [*RNQ*+], we determined the amount of soluble Rnq1 using a well-trap assay. As SDD-AGE does not allow us to consistently visualize monomeric Rnq1, the well-trap assay provided a more sensitive measure of soluble Rnq1, and hence, of how much of the Rnq1 protein is not incorporated into aggregates. In agreement with our results using SDD-AGE, we found that in cells originally propagating the s.d. very high [*RNQ*+] variant, all of the Rnq1 protein of the A4, A5, and A11 mutants was in soluble form when Sis1 was over-expressed, and could enter the SDS-PAGE gel in the fraction that was not boiled (Figure 5F). At the other end of this spectrum, all of the Rnq1 protein was in its aggregated form with the A3 and A10 mutants, thus phenocopying WT Rnq1. Mutation of the A1, A2, and A9 regions, on the other hand, resulted in an intermediate amount of soluble Rnq1 when Sis1 was over-expressed. These data show that Sis1 over-expression rescues propagation of s.d. very high [*RNQ*+] to widely varying degrees when the different amyloidogenic regions are mutated. We hypothesize that mutation of the A1, A2, and A9 regions likely allosterically impact Sis1 binding, and so are only partially rescued by Sis1 over-expression. The A3 and A10 regions, on the other hand, may represent direct binding sites for Sis1 in the context of the s.d. very high [*RNQ*+] conformation. In fact, the peptide-binding array that discovered the affinity of Sis1 for the L94 region (A3) of Rnq1 also showed some affinity, albeit weaker, for the A10 region [72]. This further supports our hypothesis that the [*RNQ*+] variants expose different parts of the Rnq1 protein to interact with Sis1.

### [*RNQ*+] variant conformation dictates dependence on Sis1 binding sites

**Figure 6:**
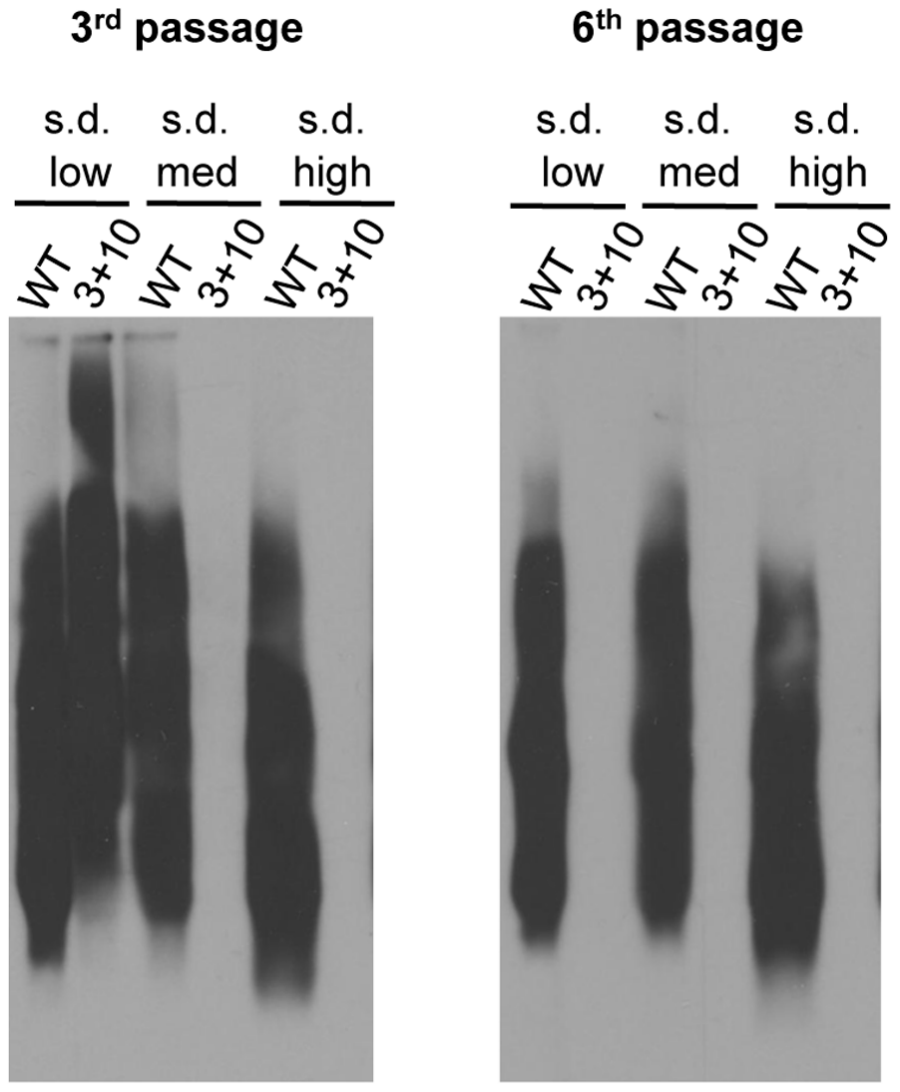
[*RNQ*+] variants rely on putative Sis1 binding sites to different extents. Propagation of s.d. high [*RNQ*+] is eliminated faster than s.d. low [*RNQ*+] when Rnq1-A3+A10 is expressed. Yeast cells propagating the indicated [*RNQ*+] variant and expressing either WT Rnq1 or Rnq1-A3+A10 were successively passaged three or six times after plasmid shuffle. Cell lysates were subjected to SDD-AGE and western blot using an αRnq1 antibody, and are representative of three independent experiments.

The data above suggest that propagation of the s.d. very high [*RNQ*+] variant likely relies on Sis1 binding to both the A3 and A10 regions. The other [*RNQ*+] variants, however, clearly show a difference in the importance of these regions for prion replication. Both the s.d. medium and m.d. high [*RNQ*+] variants were cured when the A3 region was mutated, but these conformations could still propagate with the A10 mutant (Figure 4). By contrast, mutation of neither of these regions eliminated the aggregates of s.d. low or s.d. high [*RNQ*+]. As such, we hypothesized that the A3 and A10 regions might have some redundancy with these [*RNQ*+] variants, whereby the A10 region serves as another Sis1 binding site, as our data suggest for s.d. very high [*RNQ*+], albeit to a lesser degree. To test this, we created the double mutant of Rnq1 having both the A3 and A10 regions mutated to alanine, termed Rnq1-A3+A10. We then replaced WT Rnq1 and confirmed similar expression (Figure S4). Upon continual growth, we monitored how quickly [*RNQ*+] was cured using SDD-AGE. Interestingly, mutation of both regions cured s.d. low and s.d. high [*RNQ*+], but at different rates: s.d. high [*RNQ*+] was cured much faster than s.d. low [*RNQ*+] (Figure 6). Furthermore, the double mutant cured both s.d. medium and m.d. high [*RNQ*+] faster than either single mutant (data not shown). These results indicate that both putative Sis1 binding sites in the A3 and A10 regions influence propagation of the [*RNQ*+] variants, but the extent to which these regions are utilized is variant-specific.

### Rnq1 amyloidogenic regions have [*RNQ*+] variant-dependent effects on [*PSI*+] formation

By identifying the Rnq1 amyloidogenic regions as major factors in distinguishing the ability of the [*RNQ*+] variants to propagate, we next wanted to determine whether these differences had any functional consequence on the differential ability of the [*RNQ*+] variants to induce [*PSI*+]. We envisioned two ways that these regions could affect the formation of [*PSI*+] in a variant-specific manner: 1) through mediating the differential binding of Sis1, where Sis1 plays a role in facilitating interaction with Sup35, and 2) having a different set of Rnq1 residues that facilitate the interaction with Sup35 through cross-seeding.

**Figure 7:**
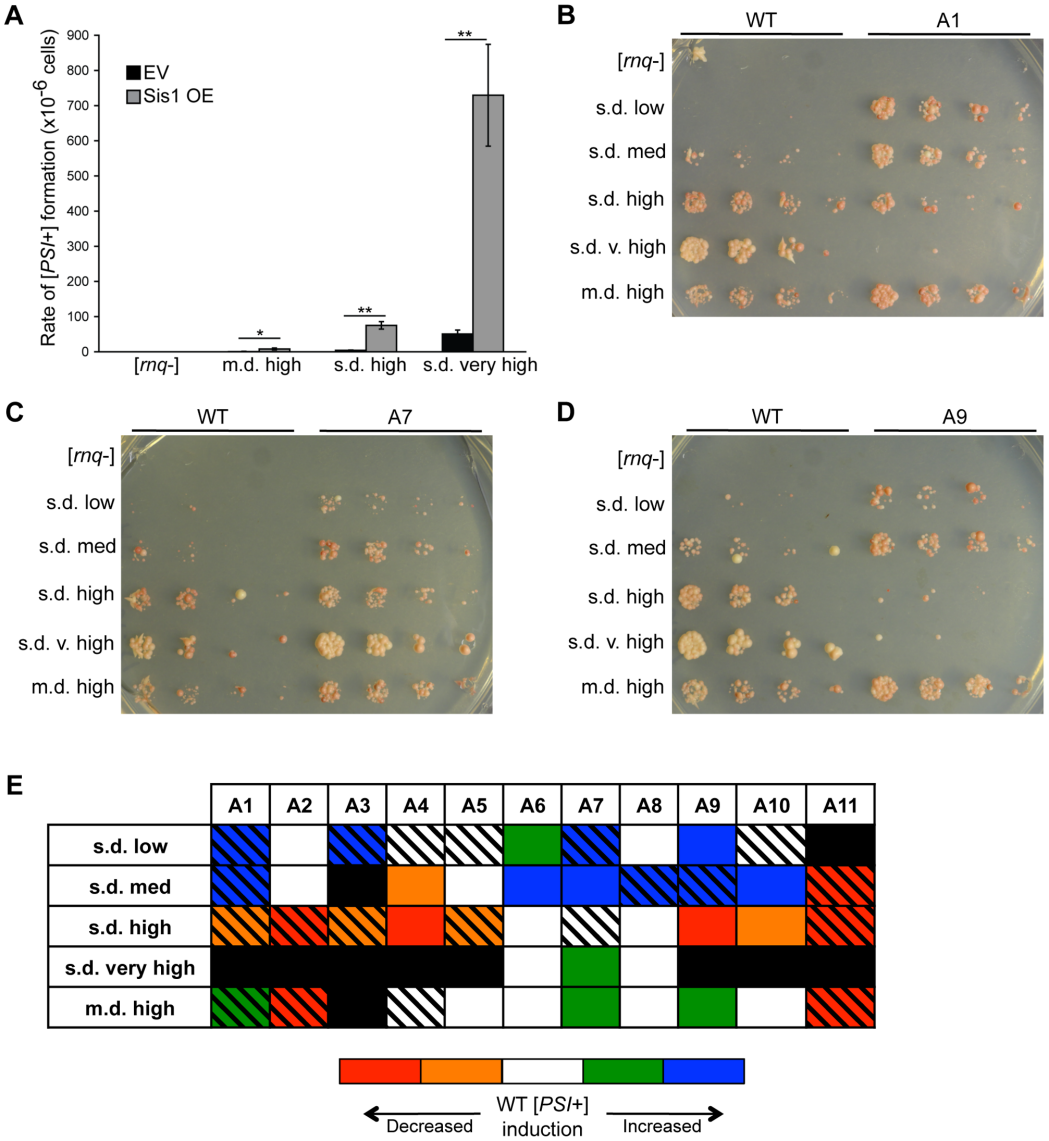
Sis1 over-expression and Rnq1 amyloidogenic regions influence the formation of [*PSI*+]. (A) Over-expression of Sis1 enhances the spontaneous formation of [*PSI*+] in [*RNQ*+] cells. [*rnq*−] [*psi*−] and [*RNQ*+] [*psi*−] cells were transformed with a Sis1 over-expressing plasmid (Sis1 OE) or an empty vector control (EV). Cultures were plated on medium that selected for [*PSI*+] cells as well as a non-selective medium to determine the total number of cells plated. Averages and error bars representing standard error of the mean were calculated from at least three independent experiments. Statistical significance was assessed using Student's *t*-test (*p<0.05, **p<0.01). (B–E) Cells expressing WT Rnq1 or the indicated mutant Rnq1 were transformed with a plasmid to over-express Sup35. [*PSI*+] induction was monitored by spotting five-fold serial dilutions of normalized cell numbers on SD-ade and SD-ade-his. Representative spottings are shown of cells expressing the mutants (B) A1, (C) A7, or (D) A9 in comparison to expression of WT Rnq1. (E) Summary of data showing how disruption of the amyloidogenic regions by alanine mutations affects [*PSI*+] induction in each of the [*RNQ*+] variants. Mutants were categorized as having no effect (white), <5-fold increase (green) or decrease (orange) in [*PSI*+] induction, or >5-fold increase (blue) or decrease (red) in [*PSI*+] induction. Black boxes indicate that [*RNQ*+] was eliminated, while black hash marks indicate that [*RNQ*+] propagation was altered (see Figure 4). Data are summarized from at least five independent experiments.

To test the first possibility, we asked whether over-expression of Sis1 would enhance the rate of spontaneous [*PSI*+] formation, as it has been shown to do for [*URE3*] [78]. The spontaneous conversion to [*PSI*+] is normally a rare event, occurring at a rate of ∼5.8×10^−7^ [79]. We transformed [*rnq*−] [*psi*−] cells or [*psi*−] cells propagating m.d. high, s.d. high, or s.d. very high [*RNQ*+] with a plasmid that over-expressed Sis1 or an empty vector control. We then selected for nonsense suppressors and screened for cells that had spontaneously converted to [*PSI*+]. We found that Sis1 over-expression significantly increased the level of spontaneous [*PSI*+] formation in [*RNQ*+] cells, but not in [*rnq*−] cells (Figure 7A). The apparent increase in [*PSI*+] formation was not due to any noticeable difference in the distribution of [*PSI*+] variants obtained (data not shown). Moreover, there were no significant differences in the levels of Hsp104 or Ssa, both of which are known to modulate the existence of [*PSI*+] (Figure S7) [80], [81]. This indicates that Sis1 helps facilitate the formation of [*PSI*+], thereby suggesting that the differential interaction between Sis1 and the [*RNQ*+] variants might contribute to their different capabilities of cross-seeding Sup35.

Next, we wanted to determine if the [*RNQ*+] variants might have different regions of Rnq1 involved in facilitating [*PSI*+] formation. Previously, we have shown that some mutations in Rnq1 that have no detectable effect on the Rnq1 aggregate structure can decrease the ability of [*RNQ*+] to induce [*PSI*+] [49]. This suggested that these residues are involved in facilitating the interaction and cross-seeding of Sup35 to form [*PSI*+]. Thus, we asked whether disruption of the Rnq1 amyloidogenic regions would show variant-specific effects on [*PSI*+] induction. In cells propagating each of the [*RNQ*+] variants and harboring the Rnq1 alanine mutants in place of WT Rnq1, we over-expressed Sup35, which greatly enhances the *de novo* formation of [*PSI*+]. We then analyzed [*PSI*+] formation by monitoring growth on media lacking adenine. Strikingly, we found that disruption of the Rnq1 amyloidogenic regions had a variety of effects on [*PSI*+] induction (Figures 7B–E and S8). As we expected, we found that some mutations that affected [*RNQ*+] also affected the formation of [*PSI*+]. For instance, mutation of the N-terminal A1 region affected propagation of all of the [*RNQ*+] variants. While we anticipated that this destabilization would cause decreased formation of [*PSI*+], it actually enhanced [*PSI*+] formation for the s.d. low, s.d. medium, and m.d. high [*RNQ*+] variants, as we saw an increase in Ade+ colonies (Figure 7B). By contrast, mutation of the A7 region had very little effect on propagation of any of the [*RNQ*+] variants, but it enhanced [*PSI*+] induction in most of them (Figure 7C). This suggests that this region might have general importance in facilitating the interaction with Sup35. Moreover, disruption of the A9 region showed quite varied effects, greatly decreasing [*PSI*+] formation with s.d. high [*RNQ*+], but increasing it with s.d. low and m.d. high [*RNQ*+] (Figure 7D), despite not grossly affecting the overall structures of these three [*RNQ*+] variants (Figure 4A). Overall, these variant-specific effects indicate that the [*RNQ*+] variants have different regions of the Rnq1 protein that are important for [*PSI*+] induction (Figure 7E). Moreover, these regions are not confined to the Rnq1-PFD.

## Discussion

It has previously been shown that a single amyloidogenic protein, Rnq1, can form a wide variety of different prion variants, many of which differentially influence the formation of [*PSI*+] [33], [44]. Similarly, it is estimated that PrP^Sc^ likely exists in over 30 different prion strains [11]. However, the physical and mechanistic basis underlying different prion strains and their associated phenotypes has only been elucidated using two variants of the [*PSI*+] prion. Our work reveals additional layers of complexity that help explain the widespread diversity of amyloid polymorphism and phenotypic variation. Here, we show *in vivo* that different amyloidogenic regions, both within and outside of the putative PFD, as well as different interactions with cofactors, are key determinants in distinguishing, and likely generating, distinct prion variants. This provides significant insight into how certain prion strains can be biochemically indistinguishable, but confer different pathological consequences [82].

Previous work has emphasized the importance of fiber stability in distinguishing prion variants [19], [20], [31], [32], [34]. The less stable Sup35 fibers of strong [*PSI*+] cause increased fragmentation into a greater number of smaller prion seeds that recruit more Sup35 monomer [20]. Similarly, less stable prion strains of PrP^Sc^ correlated with shorter incubation periods [31], [32]. In this study, we show a similar trend with the m.d. high [*RNQ*+] variant. This [*RNQ*+] variant propagated less stable Rnq1 aggregates as compared to the s.d. [*RNQ*+] variants (Figure 1A). This correlated with several properties that show that m.d. high [*RNQ*+] cells readily sequester Rnq1 monomer: solubility [44], mitotic stability, [*RRP*+] phenotype, and inviability with FL RRP (Figure 2). Hence, we propose that the decreased stability of m.d. high [*RNQ*+], like strong [*PSI*+], results in an increased number of propagons, as previously reported [52], which then enhances the ability of Rnq1 monomer to join pre-existing aggregates.

However, the decreased fiber stability does not explain all the properties of m.d. high [*RNQ*+], nor all the differences between the [*RNQ*+] variants. Despite the lower stability, which is predicted to increase fiber fragmentation into smaller aggregates, m.d. high [*RNQ*+] propagated aggregates of a larger average size than other [*RNQ*+] variants (Figure 1C). Also, both s.d. high and s.d. very high [*RNQ*+] were more thermal stable, which is predicted to cause less nonsense suppression, but these variants caused a similarly strong nonsense suppression phenotype as compared to m.d. high [*RNQ*+] (Figures 1 and 2). Therefore, our data suggest that thermal stability, while a crucial parameter, does not serve as a perfect measure of fiber fragmentation, and cannot explain the existence of many different aggregate structures, nor the large variability in phenotype. Moreover, our work and that of another group show that many additional properties that helped elucidate the physical basis of [*PSI*+] variants, similarly do not distinguish the [*RNQ*+] variants and their phenotypic effects on [*PSI*+] [33], [44], [51], [52]. These data indicate that additional factors influence prion strain propagation and the resulting phenotypes, and highlight the wide variety of polymorphic structures that can exist [7], with [*PSI*+] and [*RNQ*+] variants possibly exhibiting different types of polymorphism (e.g. packing versus segmental).

Several studies have examined the primary structural elements that are important for prion propagation, with particular emphasis on the prion-forming domains. A different length of the same stretch of contiguous residues of Sup35 was found to be protected in the amyloid structure of different [*PSI*+] variants [21]. Other work has shown that prion transmission involves the differential contribution and cooperation of discontinuous intragenic regions of the Rnq1-PFD [70]. Our data extend these findings, as we showed that disruption of certain discontinuous amyloidogenic regions in the Rnq1-PFD show variant-specific effects on [*RNQ*+] propagation (Figures 4 and S5). For instance, mutation of the A11 region, despite impairing propagation of all the [*RNQ*+] variants we analyzed, did so to varying degrees. Also, mutation of the A9 region eliminated s.d. very high [*RNQ*+] and affected propagation of s.d. medium [*RNQ*+], but had no noticeable impact on the other [*RNQ*+] variants, in agreement with some of our previous work [49]. Importantly, there were no reproducibly significant differences in protein expression of the alanine mutants that could explain their differential effects (Figure S4). Hence, these data clearly establish that the involvement and cooperation of non-adjacent regions of the PFD can vary with the type of prion variant that propagates.

In addition to the Rnq1-PFD, we found that the often-overlooked N-terminal non-Q/N-rich domain of Rnq1 has a prion variant-dependent influence on prion propagation (Figures 2 and 4). Sequences flanking the aggregation-prone part of proteins, even between regions that are far apart in the primary structure, have previously been implicated in influencing aggregation dynamics [60], [83]–[89]. For instance, amyloid fibers formed with Sup35NM as compared to those formed with full-length Sup35 have morphologically and biochemically distinct properties [90]–[92]. Similarly, it was recently shown that the region outside of the amyloid core has different structural dynamics for different [*PSI*+] variants [93]. Two models have been proposed to explain how regions outside the PFDs may be involved in amyloid assembly and propagation: 1) serving to dock monomers, with the PFD forming the amyloid core and the non-PFD outside, or 2) incorporating within the core of the fiber assembly itself [60]. Our data strongly suggest that the non-PFD domain of Rnq1 helps facilitate monomer addition into aggregates (Figure 2). However, we also show that mutation of N-terminal amyloidogenic regions differentially disrupted propagation of the [*RNQ*+] variants (Figure 4). Hence, we propose that both of these models could be correct, depending on what prion variant is propagating. In fact, as our work indicates that regions outside the unstructured Q/N-rich domains can have a major influence on propagation of particular prion variants, our data may help explain particular inconsistencies when using the full-length protein or truncated derivatives to examine the *in vivo* and *in vitro* properties of prion variants [52], [58].

Along with the influence on prion propagation, we also show that different primary structural elements likely dictate different phenotypic and pathological ramifications of prion strains. We found that mutation of certain amyloidogenic regions of Rnq1 causes variant-dependent alterations in the interaction with Sup35, thereby altering the degree of [*PSI*+] formation (Figures 7 and S8). In several cases, there was minimal to no detectable effect on maintenance of Rnq1 aggregates. Hence, while it is proposed that amino acid composition, rather than the exact sequence, is the primary driver of amyloid maintenance [94], [95], our data suggest that even relatively subtle variation in primary sequence can have profound effects on some properties of prion variants, but not others. Therefore, we have now established that the contribution of different regions of an amyloidogenic protein can help account for the biological differences seen between prion strains, such as the variation in [*PSI*+] formation mediated by the [*RNQ*+] variants.

We also discovered a striking difference in the relationship of the [*RNQ*+] variants with Sis1. Mutation of the A10 region in the Rnq1-PFD eliminated the ability of only the s.d. very high [*RNQ*+] variant to propagate (Figure 4), but this was rescued by over-expression of Sis1 (Figure 5), suggesting that the A10 region might be a secondary Sis1 binding site, in agreement with a previous peptide-binding array [72]. Interestingly, s.d. very high [*RNQ*+] was also the least affected by mutation of the known Sis1 binding site in the A3 region (using the Rnq1-L94A mutant). At the other end of this spectrum, s.d. medium [*RNQ*+] and m.d. high [*RNQ*+] were most sensitive to disruption of the A3 region, but there was no detectable effect when the A10 region was mutated. In stark contrast, for s.d. low and s.d. high [*RNQ*+], it was only when both the A3 and A10 regions were mutated that these prion variants were eliminated, with propagation of s.d. high [*RNQ*+] being abolished faster than s.d. low [*RNQ*+] (Figure 6). Surprisingly, disruption of the known Sis1 binding site using the A3 mutant did not entirely phenocopy the effects of Rnq1-L94A. We hypothesize that this might be attributed, at least in part, to higher steady state levels of Rnq1-L94A (Figure S4), and possibly in addition, how the residues surrounding L94 that are mutated in the A3 mutant are involved in propagation of particular [*RNQ*+] variants. Collectively, while we cannot definitively exclude the possibility that the Rnq1 alanine mutants are affecting multiple factors important for prion propagation, our data strongly support the hypothesis that propagation of the [*RNQ*+] variants relies on Sis1 differentially binding various sites exposed by the distinct Rnq1 aggregate structures. As we demonstrated that the over-expression of Sis1 can enhance the formation of [*PSI*+] in [*RNQ*+] cells (Figure 7A), we propose that such differential binding may influence the phenotypic impact of the [*RNQ*+] variants on [*PSI*+] inducibility.

It is suggested that similar chaperone dynamics might also influence the physical basis of [*PSI*+] variants. As Hsp104 can bind Sup35 in [*PSI*+] cells [53] and is required for propagation of the [*PSI*+] prion [80], one would predict that abrogation of the Hsp104 binding site in Sup35 would eliminate the ability of [*PSI*+] to propagate. However, propagation of a strong [*PSI*+] variant was maintained, albeit impaired, when Hsp104 binding was disrupted using *sup35Δ129-148* [96]. This suggests that Hsp104 must be able to bind other sites in Sup35. Moreover, recent work shows that this binding site can adopt different conformations for different [*PSI*+] variants, which might influence Hsp104 binding [93]. In fact, particular [*PSI*+] variants have been isolated that require increased levels of Hsp104 to stably propagate [97], [98]. Our work now provides a mechanistic explanation of these previous findings, supporting the hypothesis that different chaperone dependencies may be dictated by the exposure or conformation of different regions of the protein [99]. Indeed, these different amyloid-chaperone relationships likely explain why differences in the host environment, due to different genetic backgrounds or environmental conditions, can modulate prion propagation [100], [101]. Moreover, our data support the hypothesis that different host cofactors might interact with different amyloid structures to influence pathology [102].

With such a large variety of distinct structures that can be adopted with the same protein [6], [7], [11], [103], it was unclear how general the model that elegantly explains the physical basis of [*PSI*+] variants might be. We found that the parameters that define this model do not distinguish a set of [*RNQ*+] variants or explain their phenotypic effects. Our work now shows that disparate, non-adjacent amyloidogenic regions and complex interactions with chaperones contribute to defining the propagation of distinct prion variants. These data help elucidate what factors are involved in determining a wide array of structural differences and their associated phenotypic consequences. This may provide insight as to why some prion strains are indistinguishable based on typical biochemical properties, but have very different pathological phenotypes [82]. Furthermore, our study highlights the intricate interplay between several factors, including cofactor-amyloid interactions, which likely underlie the ability of distinct amyloid structures to co-aggregate with different proteins and cause variation in disease pathology.

## Materials and Methods

### Yeast strains and media

All yeast strains used in this study were derivatives of 74-D694 (*ade1-14 ura3-52 leu2-3,112 trp1-289 his3-Δ200*) and are described in Table S1. Yeast were grown and manipulated using standard techniques. As indicated, cells were grown in YPD (1% yeast extract, 2% peptone, 2% dextrose) or synthetic media (0.67% yeast nitrogen base without amino acids, 2% dextrose or 2% galactose+1% raffinose) lacking one or more nutrients (e.g. SD-his lacks histidine) to select for appropriate plasmids. YPD+3 mM guanidine hydrochloride (GdnHCl) was used to test prion curability. The plasmid shuffle technique was used by plating cells on media containing 1 mg/mL 5-fluoroorotic acid (5-FOA) to counterselect against cells that maintained *URA3*-containing plasmids. Reverse plasmid shuffle of WT Rnq1 was performed by transforming these Ura− cells with a *URA3* plasmid, and screening for Ura+ His- cells.

Wild-type [*rnq*−] and [*RNQ*+] yeast strains (L1751, L1943, L1945, L1767 [*psi*−], L1953, L1749) were a kind gift from Dr. Susan Liebman [44], [51]. Derivatives of these strains expressing *RRP* were constructed as described previously using pRS306-*RRP* [49]. Integration of *RRP* in the *SUP35* locus was confirmed by PCR and western blot. The *rnq1Δ::kanMX4* strains were made as described previously [49]. The *sup35Δ::hphMX4* strains were made by first passaging *Mat a* and *Mat α* 74-D694 [*PSI*+] [*RNQ*+] *sup35Δ::hphMX4* strains on YPD+3 mM GdnHCl. These were confirmed to be [*psi*−] by maintaining a red color when streaked back to YPD, and also confirmed to be [*rnq*−] by well-trap assay. These strains were then mated to *RNQ1* plasmid shuffle strains containing pRS313-*RNQ1* and propagating one of the [*RNQ*+] prion variants. Diploids were sporulated and colonies that were HygB^R^, Ura+, 5-FOA^s^, G418^s^, and His- were selected. The [*RNQ*+] variant was confirmed by well-trap assay and [*PSI*+] induction.

### Plasmid construction

All plasmids used in this study were confirmed by sequencing and are listed in Table S2, and all oligonucleotides are listed in Table S3. Construction of pRS306-*RRP*, pRS313-*RNQ1*, pRS316-*RNQ1*, pRS413*GPD-L94A*, and pEMBL-*SUP35* were described previously [33], [49], [73]. The following plasmids were gifts: pRS414*GPD-SIS1* (S. Lindquist [54]), pYK810 (M. Tuite [104]), and pYES2-*GAL-HA-RNQ1* (E. Craig [55]). Galactose-inducible *RNQ1(153-405)* was made by amplification using oligonucleotides 1626 and 0040, digestion with HindIII/XhoI, and ligation into pYES2-*GAL-HA-RNQ1*.

To create pRS315-*SUP35* and pRS315-*RRP*, the *SUP35* promoter was first amplified using oligonucleotides 1367 and 0316. This product and pRS315 were digested with SalI/BamHI and ligated to make pRS315-*SUP35p*. The *SUP35* open-reading frame and terminator were then amplified using oligonucleotides 1348 and 0322, digested with BamHI/XbaI, and ligated into pRS315-*SUP35p*. A BamHI/SacI fragment of *RRP* was digested from pRS316-*RRP* that was previously described [49] and ligated into pRS315-*SUP35p*. To make pRS315-full-length-*RRP*, full-length *RNQ1* was amplified using oligonucleotides 0477 and 0320, digested with BamHI/SacII and ligated with pRS316-*RRP*. Full-length *RRP* with the *SUP35* promoter and terminator were then amplified using oligonucleotides 1366 and 0322, digested with XhoI/SacI, and ligated into pRS315.

All *RNQ1* alanine mutants were cloned using bridge PCR. The N-terminus of *RNQ1* was amplified using oligonucleotide 0488 and the oligonucleotide containing the corresponding *rnq1* mutant. The C-terminus of *RNQ1* was amplified using the reverse complement of the mutant-specific oligonucleotide with 0489. The two amplicons were then used as a template to amplify the full-length *rnq1* mutant using oligonucleotides 0488 and 0489. This product was digested with EcoRV/SalI and ligated into the previously described pRS313 plasmid containing the *RNQ1* promoter and terminator [49], thereby expressing the Rnq1 mutants at protein levels close to WT (Figure S4). As an exception, *rnq1-A11* and the *RNQ1* terminator were amplified using oligonucleotide 0491 in place of 0489 and digested with EcoRV/XhoI. The N-terminus of *rnq1-A3+A10* was amplified using oligonucleotides 0488 and 0018 with pRS413*TPI1-rnq1-A3* as the template, while the C-terminus was amplified using oligonucleotides 0014 and 0489 with pRS313-*rnq1-A10* as the template. Similar to what we previously saw with Rnq1-L94A [73], the *rnq1* mutants *A1*, *A3*, and *A3+A10* were cloned into pRS413*TPI1* as these mutants had lower steady state protein levels as compared to WT Rnq1 when expressed from the endogenous *RNQ1* promoter. Expression from the *TPI1* promoter resulted in slightly higher protein levels for Rnq1-A1 as compared to WT Rnq1, but WT protein levels for Rnq1-A3 and Rnq1-A3+A10 (Figure S4). Furthermore, the A1, A2, and A11 mutants consistently ran higher than WT Rnq1 by SDS-PAGE despite having the same number of amino acids. To make pRS413*TPI1*, the *TPI1* promoter was amplified using oligonucleotides 1429 and 1430, digested with SacI/XbaI, and ligated into pRS413*ADH* cut with the same enzymes to replace the *ADH1* promoter. Finally, pRS413*TEF-RNQ1(132-405)* was cloned with oligonucleotides 1436 and 0489 and digested with EcoRV/SalI.

### Protein analysis

Sedimentation of Rnq1 by solubility assay and SDD-AGE were performed using established methods [33], [49]. For well-trap and thermal stability assays, yeast cell lysates were prepared by vortexing with glass beads in buffer (100 mM Tris pH 7.5, 200 mM NaCl, 1 mM EDTA, 5% glycerol, 0.5 mM DTT, 3 mM PMSF, 50 mM N-Ethylmaleimide (NEM), complete protease inhibitor from Roche). Lysates were pre-cleared by centrifugation at 3,300*g* for 15 sec. For well-trap assays, protein concentration was normalized, and samples were treated in sample buffer (50 mM Tris-HCl pH 6.8, 10% glycerol, 2% SDS, 100 mM DTT) for 5 minutes at room temperature or 100°C. For thermal stability assays, samples were also incubated at a temperature gradient (45°C to 95°C) for 5 minutes. Samples were then analyzed by SDS-PAGE and western blot using a polyclonal αRnq1 antibody.

### Limited proteolysis

[*RNQ*+] cell lysates were prepared as above for well-trap assays, with the exception of adding PMSF to the buffer. Protein concentration was normalized to ∼3 µg/µl, followed by addition of proteinase K (Sigma-Aldrich) to varying concentrations. Samples were incubated at 37°C in a water bath for 30 min or 1 hr as indicated. The reaction was stopped by adding sample buffer and boiling samples at 100°C for 5 min, followed by SDS-PAGE and western blot using an αRnq1 antibody. Cell lysates propagating the m.d. high [*RNQ*+] variant consistently showed higher starting concentrations of the Rnq1 protein, likely because the very thermal stable Rnq1 aggregates of s.d. [*RNQ*+] were not completely broken down.

### Immunoprecipitation

For co-immunoprecipitation of Sis1 and Rnq1, cultures were grown overnight, washed, and lysed by vortexing in buffer (50 mM Tris pH 8, 150 mM NaCl, 1 mM EDTA, 0.2% Triton X-100, 1 mM PMSF, 50 mM NEM, complete protease inhibitor from Roche) containing glass beads. Lysates were pre-cleared by centrifugation at 9,300*g* for 30 sec at 4°C. Protein was normalized to 1 mg/mL. Following incubation with or without αSis1 antibodies for 1 hr at 4°C, Protein G Sepharose (GE Healthcare) beads were added and incubated for 1 hr at 4°C. Beads were pelleted by centrifugation at 800*g* for 30 sec, followed by washing three times, and boiling at 100°C for 5 min. Samples were analyzed by SDS-PAGE and western blot using αRnq1 and αSis1 antibodies.

### Phenotypic colorimetric assays

Taking advantage of the ability of [*RNQ*+] cells harboring *RRP* to suppress *ade1-14*, we monitored [*RRP*+] phenotypes as done previously [33]. Equal numbers of cells were normalized by OD_600_, serially diluted five-fold, and spotted to the indicated media. SD-ade plates were incubated at 30°C for 6 days, while all other types of media were incubated for 3 days, followed by overnight at 4°C for color development. Mitotic stability assays were performed as before with at least three independent cultures for each [*RNQ*+] variant [34].

### Joining assay

Cells were transformed with pYES2-*GAL-HA-RNQ1* or pYES2-*GAL-RNQ1(153-405)*. Overnight cultures grown in glucose-based selection media were washed and diluted to OD_600_ 0.3 in induction media containing 2% galactose+1% raffinose, followed by harvesting after 3 hrs of growth. Cell lysates were prepared and analyzed by well-trap assay.

### [*PSI*+] formation

To monitor the spontaneous conversion to [*PSI*+], cells were first transformed with pRS414*GPD-SIS1* or an empty vector control. Then, using suppression of *ade1-14* to monitor [*PSI*+] formation, at least three independent cultures were grown overnight and 200 µl of culture was plated on SD-ade-trp and 200 µl of a 1∶10,000 dilution was plated on SD-trp. SD-ade-trp plates were grown overnight at 30°C, followed by two weeks at 4°C, then two weeks at 30°C, as it has been reported that cold enhances prion formation [40]. To calculate the number of cells plated on SD-ade-trp, the colonies on SD-trp were counted, averaged, and multiplied by the dilution factor of 10,000. Over 7.1×10^6^ cells were plated on SD-ade-trp for each condition. Ade+ colonies were then scored as [*PSI*+] by spotting to YPD and SD-ade, as well as YPD+3 mM GdnHCl to confirm curability. The rate of [*PSI*+] formation was then calculated as the ratio of [*PSI*+] colonies to the total number of cells plated.

The induction of [*PSI*+] by over-expression of Sup35 was performed as previously described [33]. Briefly, cells expressing the indicated Rnq1 construct were transformed with pEMBL-*SUP35*. Overnight cultures were normalized by OD_600_ and spotted in five-fold serial dilutions to SD-his, SD-ade, and SD-ade-his. At least five independent experiments were performed. As previously reported, over 88% of colonies are bona fide [*PSI*+] [49].

## Supporting Information

Figure S1 Differential proteinase K resistance of Rnq1 in [*RNQ*+] variants. Rnq1 aggregates of the m.d. high [*RNQ*+] variant reproducibly show enhanced protease resistance as compared to s.d. [*RNQ*+] variants. Lysates of cells propagating the indicated [*RNQ*+] variant were incubated with a gradient of different proteinase K (PK) concentrations at 37°C for 30 min, followed by SDS-PAGE and western blot analysis using an αRnq1 antibody.
(TIF)

Figure S2 Inviability of FL RRP in m.d. high [*RNQ*+] cells depends on Rnq1 expression. Cultures of three different clones of *rnq1Δ* cells propagating m.d. high [*RNQ*+], along with controls of [*rnq*−] cells, cells cured of m.d. high [*RNQ*+], and m.d. high [*RNQ*+] cells, all expressing FL RRP were normalized by OD_600_, serially diluted five-fold, and spotted on media to select for loss (− Sup35) or co-expression (+ Sup35) of wild-type Sup35. Representative spottings are shown.
(TIF)

Figure S3 Expression of FL RRP in cells propagating s.d. [*RNQ*+] variants. Cultures of [*rnq*−] cells or cells propagating s.d. low, s.d. medium, or s.d. very high [*RNQ*+] and expressing Sup35, RRP, or full-length (FL) RRP were normalized by OD_600_, serially diluted five-fold, and spotted to select for loss (− Sup35) or co-expression (+ Sup35) of wild-type Sup35. Representative spottings are shown.
(TIF)

Figure S4 Protein expression of Rnq1 alanine mutants. Normalized protein from cell lysates expressing the indicated Rnq1 alanine mutant, or an empty vector (EV), in place of WT Rnq1, was subjected to SDS-PAGE and western blot using an αRnq1 antibody.
(TIF)

Figure S5 Summary of effects on [*RNQ*+] propagation by mutation of Rnq1 amyloidogenic regions according to assay. For the indicated assay, mutants are categorized as having no effect (white), mild effect (yellow), moderate effect (orange), or not propagating [*RNQ*+] (red) after successive passaging. (A) Examples of how the Rnq1 solubility assay was scored. Cells propagating m.d. high [*RNQ*+] (m.d.), s.d. low [*RNQ*+] (Low), or s.d. very high [*RNQ*+] (VH), and harboring either wild-type (WT) Rnq1 or the indicated Rnq1 alanine mutant, were fractionated by high-speed ultracentrifugation into total (T), supernatant (S), and pellet (P) fractions, followed by SDS-PAGE and western blot using an αRnq1 antibody. Mutants were characterized as follows: 1) mild effect, if there was a slight increase in the amount of soluble Rnq1; 2) moderate effect, if the supernatant and pellet fractions contained roughly equal amounts of Rnq1; or 3) cured, if all of Rnq1 accumulated in the supernatant. (B) Examples of how the thermal stability of Rnq1 aggregates was scored. Cells propagating the indicated [*RNQ*+] variant with either WT Rnq1 or the indicated Rnq1 alanine mutant were lysed and treated with a temperature gradient, followed by SDS-PAGE and western blot using an αRnq1 antibody. Mutants were characterized as follows: 1) mild effect, if there was a slight, but reproducible shift in the more intense bands; 2) moderate effect, if the number of intense bands shifted by four or more lanes; 3) cured, if there was roughly equal amounts of Rnq1 in each lane, which was confirmed by well-trap assay; or 4) more stable, if Rnq1 was reproducibly present in fewer lanes. (C) Summary table of the effects of Rnq1 mutants on [*RNQ*+] propagation. Unless the phenotypic change is noted, colors indicate: decreased aggregate densitometry (SDD-AGE), decreased stability (Thermal stability), or increased pool of soluble Rnq1 (Solubility assay). Data are summarized from at least three independent experiments.
(TIF)

Figure S6 Disruption of Rnq1 amyloidogenic regions eliminates propagation of [*RNQ*+] variants. Cells originally propagating the indicated [*RNQ*+] variant and expressing WT Rnq1 or a Rnq1 alanine mutant from a *HIS*-marked plasmid (as in Figure 4) were transformed with a *RNQ1 URA3*-marked plasmid. These cells were then screened for loss of the indicated Rnq1 construct in order to express WT Rnq1 from the *URA3*-marked plasmid as the only copy of Rnq1. For instance, P-WT and P-A3 refer to post (P) expression of the *HIS*-marked copy of WT Rnq1 and Rnq1-A3, respectively, and now both expressing WT Rnq1. The presence of Rnq1 aggregates in these Ura+ his- cells was then monitored using SDD-AGE and western blot using an αRnq1 antibody.
(TIF)

Figure S7 Expression levels of Ssa and Hsp104 are unchanged when Sis1 is over-expressed. Cells with the indicated [*RNQ*+] status were transformed with a Sis1 over-expressing plasmid (+) or an empty vector control (−). Lysates were subjected to SDS-PAGE and western blot using αHsp104, αHsp70-Ssa, αSis1, and αPgk1 antibodies.
(TIF)

Figure S8 Formation of [*PSI*+] is altered by mutation of Rnq1 amyloidogenic regions. [*rnq*−] cells or cells propagating the indicated [*RNQ*+] variant and expressing WT Rnq1 or mutants (A) A2, (B) A3, (C) A4, (D) A5, (E) A6, (F) A8, (G) A10, or (H) A11 were transformed with a plasmid over-expressing Sup35. [*PSI*+] induction was monitored by spotting five-fold serial dilutions of normalized numbers of cells on SD-ade and SD-ade-his. Representative spottings from at least five independent experiments are shown.
(TIF)

Table S1 Yeast strains used in this study.
(DOC)

Table S2 Plasmids used in this study.
(DOC)

Table S3 Oligonucleotides used in this study.
(DOC)
