# Supplementary figures and images for: Extensive Diversity of Prion Strains Is Defined by Differential Chaperone Interactions and Distinct Amyloidogenic Regions
Source: PLoS Genet. 2014 May 8;10(5):e1004337. doi: 10.1371/journal.pgen.1004337 (PMC4014422; doi:10.1371/journal.pgen.1004337)

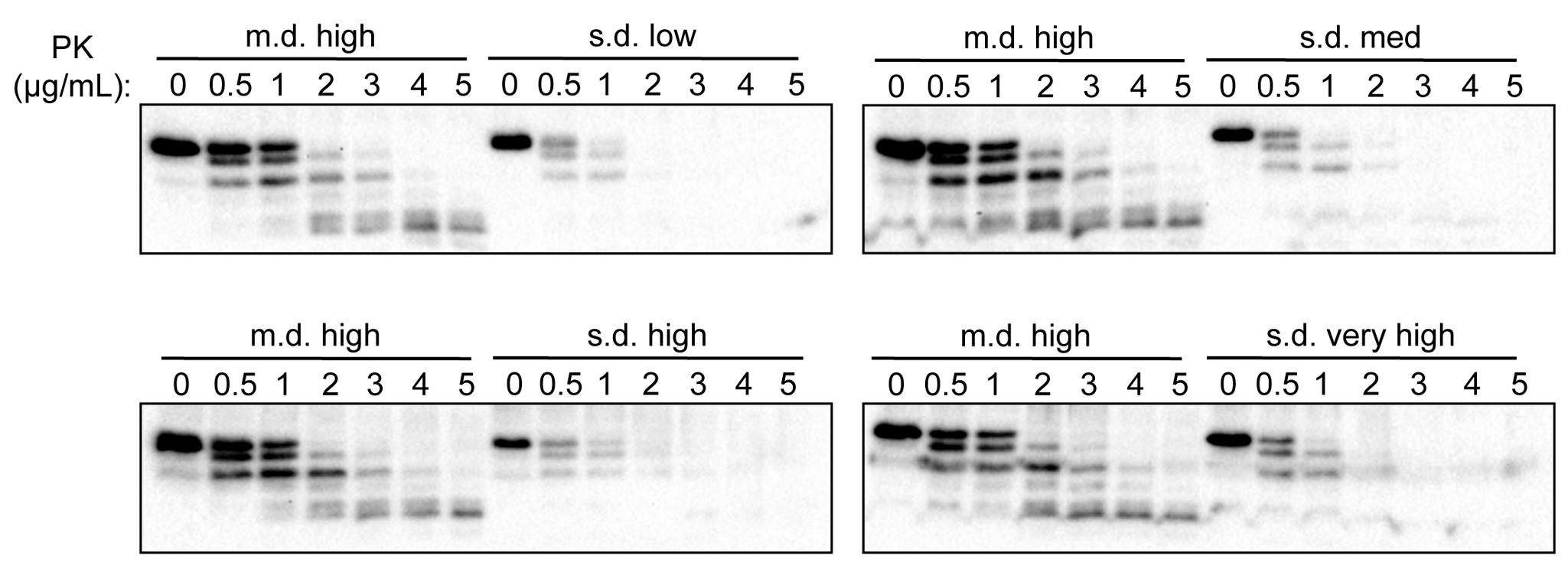

Supplement: Figure S1 — Differential proteinase K resistance of Rnq1 in [RNQ+] variants. Rnq1 aggregates of the m.d. high [RNQ+] variant reproducibly show enhanced protease resistance as compared to s.d. [RNQ+] variants. Lysates of cells propagating the indicated [RNQ+] variant were incubated with a gradient of different proteinase K (PK) concentrations at 37°C for 30 min, followed by SDS-PAGE and western blot analysis using an αRnq1 antibody. (TIF) [file pgen.1004337.s001.tif]

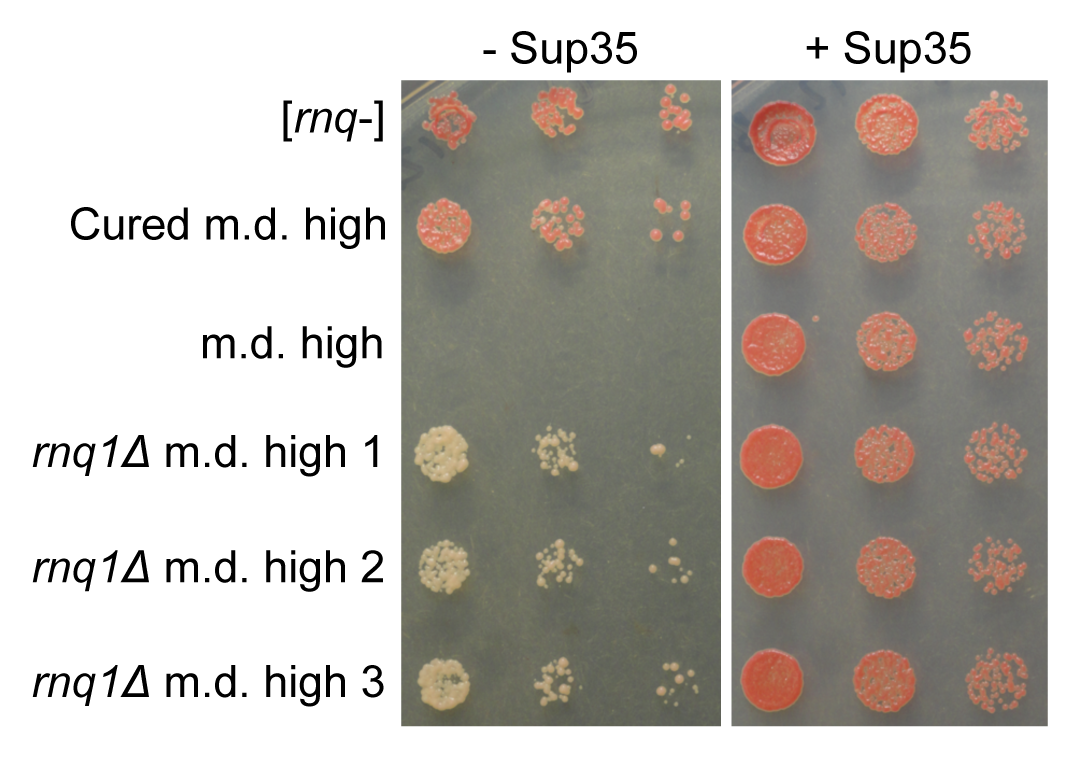

Supplement: Figure S2 — Inviability of FL RRP in m.d. high [RNQ+] cells depends on Rnq1 expression. Cultures of three different clones of rnq1Δ cells propagating m.d. high [RNQ+], along with controls of [rnq−] cells, cells cured of m.d. high [RNQ+], and m.d. high [RNQ+] cells, all expressing FL RRP were normalized by OD600, serially diluted five-fold, and spotted on media to select for loss (− Sup35) or co-expression (+ Sup35) of wild-type Sup35. Representative spottings are shown. (TIF) [file pgen.1004337.s002.tif]

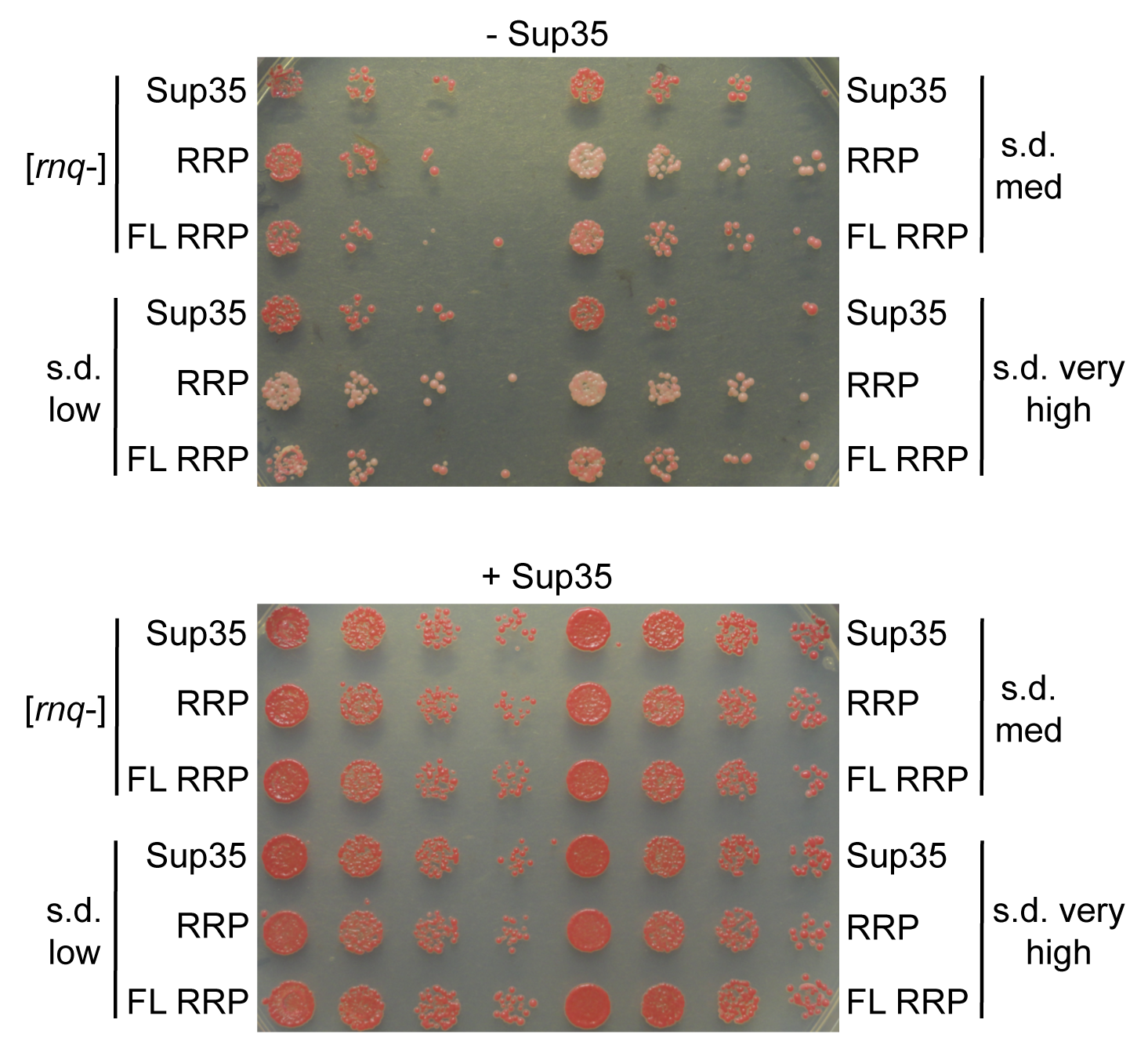

Supplement: Figure S3 — Expression of FL RRP in cells propagating s.d. [RNQ+] variants. Cultures of [rnq−] cells or cells propagating s.d. low, s.d. medium, or s.d. very high [RNQ+] and expressing Sup35, RRP, or full-length (FL) RRP were normalized by OD600, serially diluted five-fold, and spotted to select for loss (− Sup35) or co-expression (+ Sup35) of wild-type Sup35. Representative spottings are shown. (TIF) [file pgen.1004337.s003.tif]

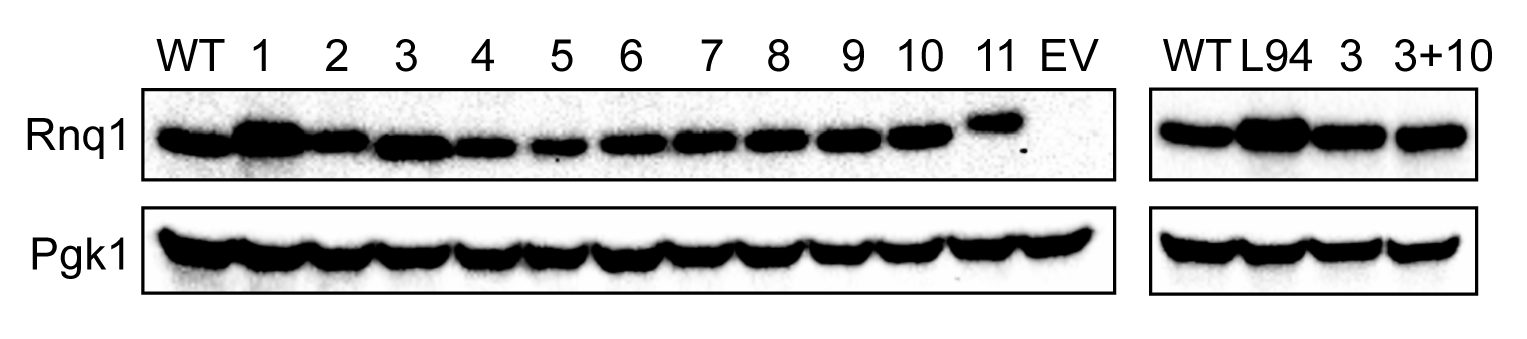

Supplement: Figure S4 — Protein expression of Rnq1 alanine mutants. Normalized protein from cell lysates expressing the indicated Rnq1 alanine mutant, or an empty vector (EV), in place of WT Rnq1, was subjected to SDS-PAGE and western blot using an αRnq1 antibody. (TIF) [file pgen.1004337.s004.tif]

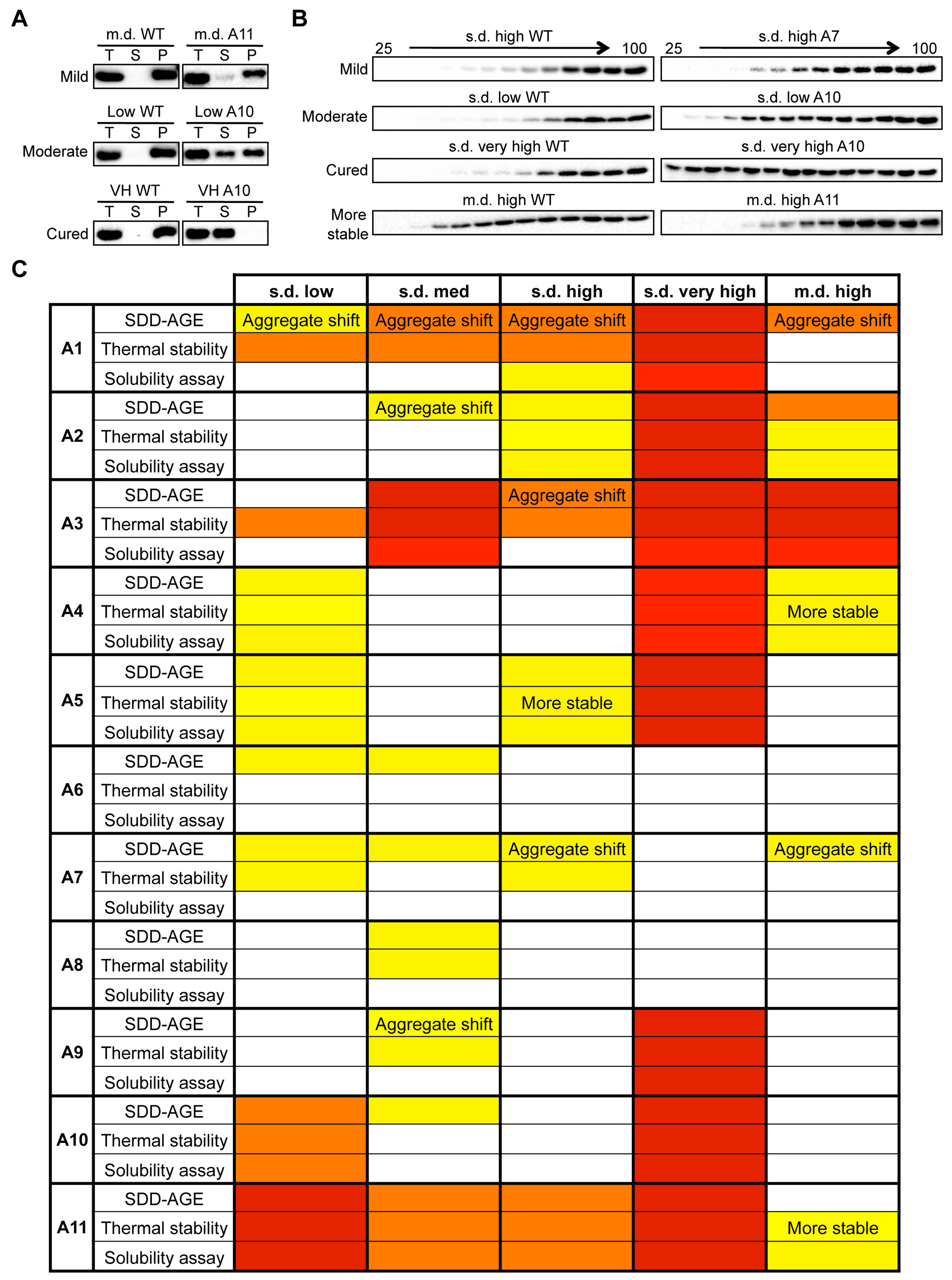

Supplement: Figure S5 — Summary of effects on [RNQ+] propagation by mutation of Rnq1 amyloidogenic regions according to assay. For the indicated assay, mutants are categorized as having no effect (white), mild effect (yellow), moderate effect (orange), or not propagating [RNQ+] (red) after successive passaging. (A) Examples of how the Rnq1 solubility assay was scored. Cells propagating m.d. high [RNQ+] (m.d.), s.d. low [RNQ+] (Low), or s.d. very high [RNQ+] (VH), and harboring either wild-type (WT) Rnq1 or the indicated Rnq1 alanine mutant, were fractionated by high-speed ultracentrifugation into total (T), supernatant (S), and pellet (P) fractions, followed by SDS-PAGE and western blot using an αRnq1 antibody. Mutants were characterized as follows: 1) mild effect, if there was a slight increase in the amount of soluble Rnq1; 2) moderate effect, if the supernatant and pellet fractions contained roughly equal amounts of Rnq1; or 3) cured, if all of Rnq1 accumulated in the supernatant. (B) Examples of how the thermal stability of Rnq1 aggregates was scored. Cells propagating the indicated [RNQ+] variant with either WT Rnq1 or the indicated Rnq1 alanine mutant were lysed and treated with a temperature gradient, followed by SDS-PAGE and western blot using an αRnq1 antibody. Mutants were characterized as follows: 1) mild effect, if there was a slight, but reproducible shift in the more intense bands; 2) moderate effect, if the number of intense bands shifted by four or more lanes; 3) cured, if there was roughly equal amounts of Rnq1 in each lane, which was confirmed by well-trap assay; or 4) more stable, if Rnq1 was reproducibly present in fewer lanes. (C) Summary table of the effects of Rnq1 mutants on [RNQ+] propagation. Unless the phenotypic change is noted, colors indicate: decreased aggregate densitometry (SDD-AGE), decreased stability (Thermal stability), or increased pool of soluble Rnq1 (Solubility assay). Data are summarized from at least three independent experiments. (TIF) [file pgen.1004337.s005.tif]

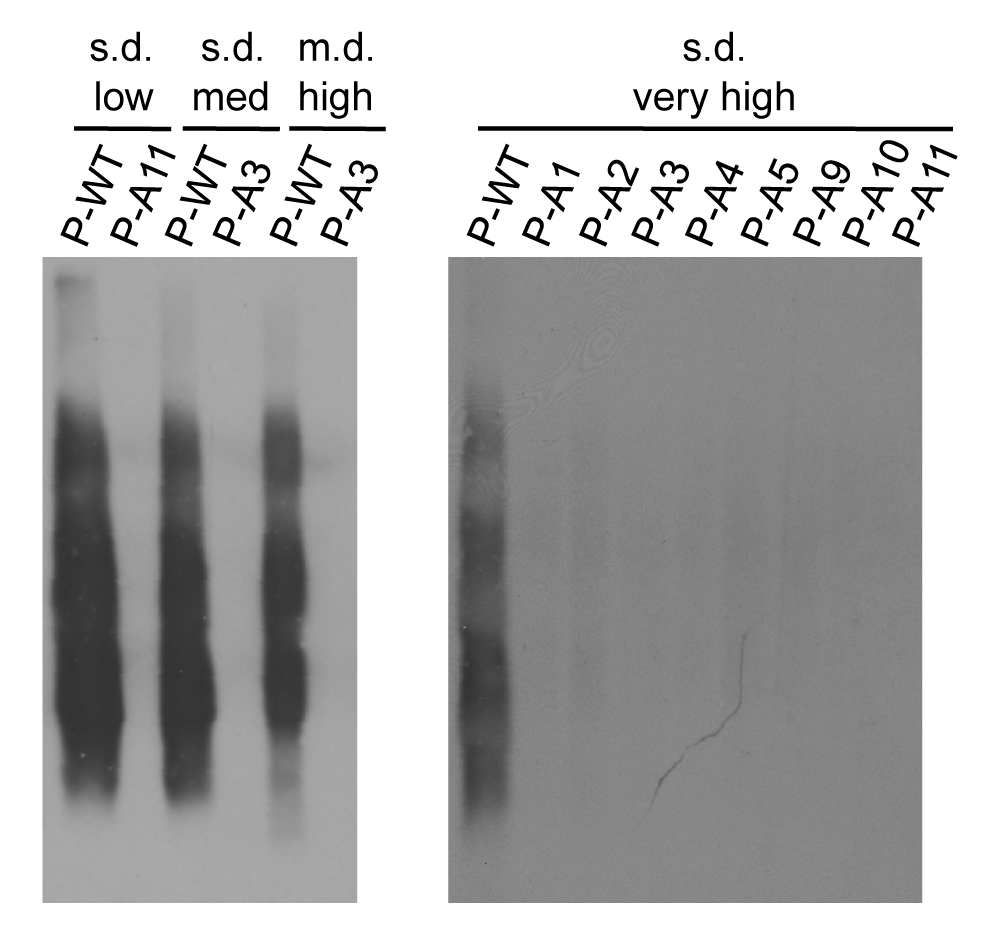

Supplement: Figure S6 — Disruption of Rnq1 amyloidogenic regions eliminates propagation of [RNQ+] variants. Cells originally propagating the indicated [RNQ+] variant and expressing WT Rnq1 or a Rnq1 alanine mutant from a HIS-marked plasmid (as in Figure 4) were transformed with a RNQ1 URA3-marked plasmid. These cells were then screened for loss of the indicated Rnq1 construct in order to express WT Rnq1 from the URA3-marked plasmid as the only copy of Rnq1. For instance, P-WT and P-A3 refer to post (P) expression of the HIS-marked copy of WT Rnq1 and Rnq1-A3, respectively, and now both expressing WT Rnq1. The presence of Rnq1 aggregates in these Ura+ his- cells was then monitored using SDD-AGE and western blot using an αRnq1 antibody. (TIF) [file pgen.1004337.s006.tif]

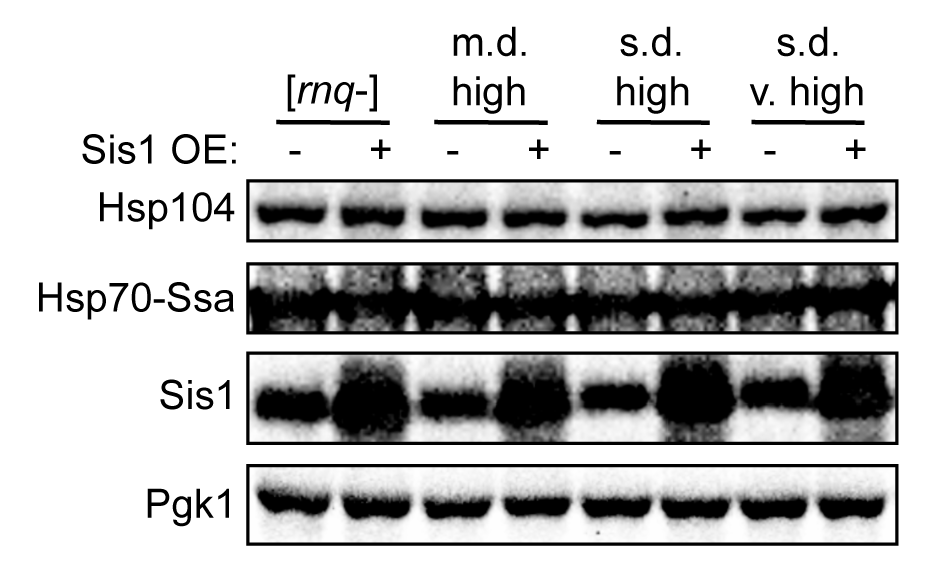

Supplement: Figure S7 — Expression levels of Ssa and Hsp104 are unchanged when Sis1 is over-expressed. Cells with the indicated [RNQ+] status were transformed with a Sis1 over-expressing plasmid (+) or an empty vector control (−). Lysates were subjected to SDS-PAGE and western blot using αHsp104, αHsp70-Ssa, αSis1, and αPgk1 antibodies. (TIF) [file pgen.1004337.s007.tif]

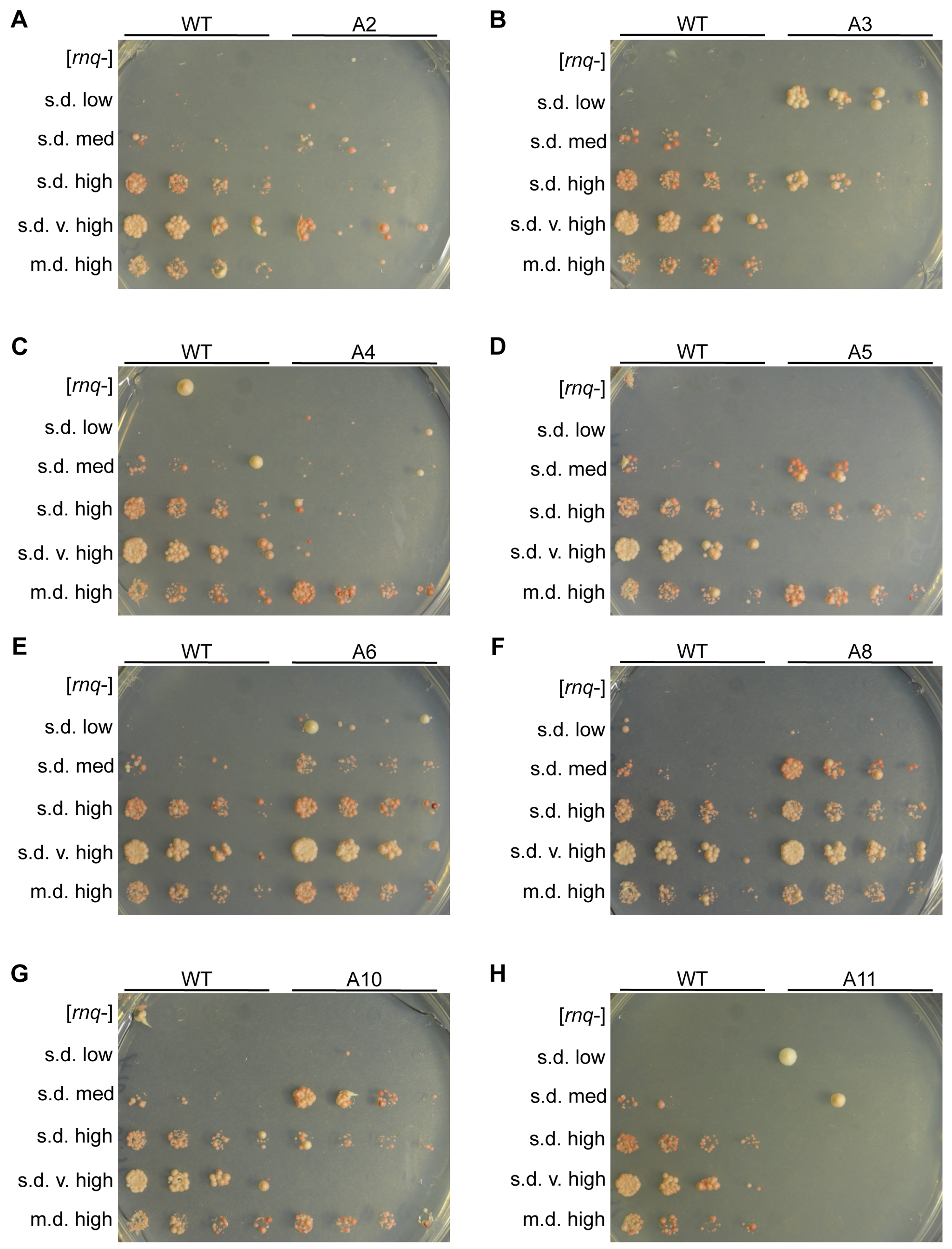

Supplement: Figure S8 — Formation of [PSI+] is altered by mutation of Rnq1 amyloidogenic regions. [rnq−] cells or cells propagating the indicated [RNQ+] variant and expressing WT Rnq1 or mutants (A) A2, (B) A3, (C) A4, (D) A5, (E) A6, (F) A8, (G) A10, or (H) A11 were transformed with a plasmid over-expressing Sup35. [PSI+] induction was monitored by spotting five-fold serial dilutions of normalized numbers of cells on SD-ade and SD-ade-his. Representative spottings from at least five independent experiments are shown. (TIF) [file pgen.1004337.s008.tif]
