# Supplementary material for: Extensive Diversity of Prion Strains Is Defined by Differential Chaperone Interactions and Distinct Amyloidogenic Regions
Source: PLoS Genet. 2014 May 8;10(5):e1004337. doi: 10.1371/journal.pgen.1004337 (PMC4014422; doi:10.1371/journal.pgen.1004337)
Supplement: Table S1 — Yeast strains used in this study. (DOC) [file pgen.1004337.s009.doc]

Table S1. Yeast strains used in this study.

| **Strain** | **Description** | **Reference** |
| --- | --- | --- |
| 1060 | *Mat a ade1-14 ura3-52 leu2-3,112 trp1-289 his3-Δ200 sup35Δ::RMC* [*rnq*-] | [49] |
| 693 | *Mat a ade1-14 ura3-52 leu2-3,112 trp1-289 his3-Δ200 sup35Δ::RMC* s.d. low [*RNQ*+] | [34] |
| 694 | *Mat a ade1-14 ura3-52 leu2-3,112 trp1-289 his3-Δ200 sup35Δ::RMC* s.d. medium [*RNQ*+] | [34] |
| 2311 | *Mat a ade1-14 ura3-52 leu2-3,112 trp1-289 his3-Δ200 sup35Δ::RMC* s.d. high [*RNQ*+] | This study |
| 2042 | *Mat a ade1-14 ura3-52 leu2-3,112 trp1-289 his3-Δ200 sup35Δ::RMC* s.d. very high [*RNQ*+] | This study |
| 459 | *Mat a ade1-14 ura3-52 leu2-3,112 trp1-289 his3-Δ200 sup35Δ::RMC* m.d. high [*RNQ*+] | [34,49] |
| 2043 | *Mat a ade1-14 ura3-52 leu2-3,112 trp1-289 his3-Δ200 sup35Δ::RMC rnq1Δ::kanMX4* [*rnq*-] | This study |
| 2044 | *Mat a ade1-14 ura3-52 leu2-3,112 trp1-289 his3-Δ200 sup35Δ::RMC rnq1Δ::kanMX4* s.d. low [*RNQ*+] | This study |
| 2045 | *Mat a ade1-14 ura3-52 leu2-3,112 trp1-289 his3-Δ200 sup35Δ::RMC rnq1Δ::kanMX4* s.d. medium [*RNQ*+] | This study |
| 2369 | *Mat α ade1-14 ura3-52 leu2-3,112 trp1-289 his3-Δ200 sup35Δ::RMC rnq1Δ::kanMX4* s.d. high [*RNQ*+] | This study |
| 2053 | *Mat a ade1-14 ura3-52 leu2-3,112 trp1-289 his3-Δ200 sup35Δ::RMC rnq1Δ::kanMX4* s.d. very high [*RNQ*+] | This study |
| 2047 | *Mat a ade1-14 ura3-52 leu2-3,112 trp1-289 his3-Δ200 sup35Δ::RMC rnq1Δ::kanMX4* m.d. high [*RNQ*+] | This study |
| 1890 | *Mat a ade1-14 ura3-52 leu2-3,112 trp1-289 his3-Δ200 sup35Δ::hphMX4* [*psi*-] [*rnq*-] | This study |
| 2259 | *Mat a ade1-14 ura3-52 leu2-3,112 trp1-289 his3-Δ200 sup35Δ::hphMX4* [*psi*-] s.d. low [*RNQ*+] | This study |
| 2260 | *Mat a ade1-14 ura3-52 leu2-3,112 trp1-289 his3-Δ200 sup35Δ::hphMX4* [*psi*-] s.d. medium [*RNQ*+] | This study |
| 2416 | *Mat α ade1-14 ura3-52 leu2-3,112 trp1-289 his3-Δ200 sup35Δ::hphMX4* [*psi*-] s.d. high [*RNQ*+] | This study |
| 2262 | *Mat a ade1-14 ura3-52 leu2-3,112 trp1-289 his3-Δ200 sup35Δ::hphMX4* [*psi*-] s.d. very high [*RNQ*+] | This study |
| 2261 | *Mat α ade1-14 ura3-52 leu2-3,112 trp1-289 his3-Δ200 sup35Δ::hphMX4* [*psi*-] m.d. high [*RNQ*+] | This study |
| 2055 | *Mat a ade1-14 ura3-52 leu2-3,112 trp1-289 his3-Δ200 rnq1Δ::kanMX4* [*psi*-] [*rnq*-] | This study |
| 831 | *Mat α ade1-14 ura3-52 leu2-3,112 trp1-289 his3-Δ200 rnq1Δ::kanMX4* [*psi*-] s.d. low [*RNQ*+] | [49] |
| 833 | *Mat α ade1-14 ura3-52 leu2-3,112 trp1-289 his3-Δ200 rnq1Δ::kanMX4* [*psi*-] s.d. medium [*RNQ*+] | [49] |
| 2310 | *Mat a ade1-14 ura3-52 leu2-3,112 trp1-289 his3-Δ200 rnq1Δ::kanMX4* [*psi*-] s.d. high [*RNQ*+] | This study |
| 2057 | *Mat a ade1-14 ura3-52 leu2-3,112 trp1-289 his3-Δ200 rnq1Δ::kanMX4* [*psi*-] s.d. very high [*RNQ*+] | This study |
| 835 | *Mat α ade1-14 ura3-52 leu2-3,112 trp1-289 his3-Δ200 rnq1Δ::kanMX4* [*psi*-] m.d. high [*RNQ*+] | [49] |
| L1751 | *Mat a ade1-14 ura3-52 leu2-3,112 trp1-289 his3-Δ200* [*psi*-] [*rnq*-] | [44] |
| L1943 | *Mat a ade1-14 ura3-52 leu2-3,112 trp1-289 his3-Δ200* [*psi*-] s.d. low [*RNQ*+] | [44] |
| L1945 | *Mat a ade1-14 ura3-52 leu2-3,112 trp1-289 his3-Δ200* [*psi*-] s.d. medium [*RNQ*+] | [44] |
| L1767 [*psi*-] | *Mat a ade1-14 ura3-52 leu2-3,112 trp1-289 his3-Δ200* [*psi*-] s.d. high [*RNQ*+] | [51] |
| L1953 | *Mat a ade1-14 ura3-52 leu2-3,112 trp1-289 his3-Δ200* [*psi*-] s.d. very high [*RNQ*+] | [44] |
| L1749 | *Mat a ade1-14 ura3-52 leu2-3,112 trp1-289 his3-Δ200* [*psi*-] m.d. high [*RNQ*+] | [44] |
