# Supplementary material for: Extensive Diversity of Prion Strains Is Defined by Differential Chaperone Interactions and Distinct Amyloidogenic Regions
Source: PLoS Genet. 2014 May 8;10(5):e1004337. doi: 10.1371/journal.pgen.1004337 (PMC4014422; doi:10.1371/journal.pgen.1004337)
Supplement: Table S2 — Plasmids used in this study. (DOC) [file pgen.1004337.s010.doc]

Table S2. Plasmids used in this study.

| **Plasmid** | **Description** | **Reference** |
| --- | --- | --- |
| 5245 | pRS306-*RRP* | [49] |
| 6246 | pRS315*-SUP35* | This study |
| 6501 | pRS315-*RRP* | This study |
| 6479 | pRS315-full-length-*RRP* | This study |
| SL6602 | pRS414*GPD-SIS1* | [54] |
| 6244 | pEMBL-*SUP35* | This study |
| 5908 | p413*GPD*-*rnq1-L94A* | [73] |
| 6312 | pRS313-*RNQ1* | [49] |
| 6352 | pRS413*TPI1-rnq1-A1* | This study |
| 6392 | pRS313-*rnq1-A2* | This study |
| 6389 | pRS413*TPI1-rnq1-A3* | This study |
| 6322 | pRS313-*rnq1-A4* | This study |
| 6411 | pRS313-*rnq1-A5* | This study |
| 6477 | pRS313-*rnq1-A6* | This study |
| 6478 | pRS313-*rnq1-A7* | This study |
| 6397 | pRS313-*rnq1-A8* | This study |
| 6165 | pRS313-*rnq1-A9* | This study |
| 6398 | pRS313-*rnq1-A10* | This study |
| 6416 | pRS313-*rnq1-A11* | This study |
| 6548 | pRS413*TPI1-rnq1-A3+A10* | This study |
| 6357 | pRS413*TEF-RNQ1(132-405)* | This study |
| 5195 | pRS316-*RNQ1* | [49] |
| 5049 | pYK810 | [104] |
| 6545 | pYES2-*GAL-HA-RNQ1* | [55] |
| 6613 | pYES2-*GAL-RNQ1(153-405)* | This study |
