# Supplementary material for: Extensive Diversity of Prion Strains Is Defined by Differential Chaperone Interactions and Distinct Amyloidogenic Regions
Source: PLoS Genet. 2014 May 8;10(5):e1004337. doi: 10.1371/journal.pgen.1004337 (PMC4014422; doi:10.1371/journal.pgen.1004337)
Supplement: Table S3 — Oligonucleotides used in this study. (DOC) [file pgen.1004337.s011.doc]

Table S3. Oligonucleotides used in this study.

| **Oligonucleotide** | **Description** | **Sequence** |
| --- | --- | --- |
| 1366 | 5’ Sup35prom-XhoI | 5'CGCCTCGAGGACGACGCGTCACAGTG |
| 1367 | 5’ Sup35prom-SalI | 5'CGCGTCGACGACGACGCGTCACAGTG |
| 0316 | 3’Sup35prom-BamHI | 5'CCCGGATCCTGTTGCTAGTGGGCAGATATAG |
| 1348 | 5’ Sup35-BamHI-SpeI | 5'CGCGGATCCACTAGTATGTCGGATTCAAACCAAGG |
| 0322 | 3’Sup35(124-685)term-SacI | 5’GGGGAGCTCGTGATTGAAGGAGTTGAAACCTTGC |
| 0014 | 5'-TO-RNQ-4 | 5'GGTGGCAACGGTGGTTACC |
| 0018 | 3'-TO-RNQ-8 | 5'AAAGCACCACCAGATTGTCC |
| 0477 | 5’ Rnq1-BamHI | 5'CCCGGATCCATGGATACGGATAAGTTAATCTCAGAGG |
| 0320 | 3’RNQ1(153-405)-SacII | 5'GGGCCGCGGGTAGCGGTTCTGGTTGCCGTTATTG |
| 0488 | 5' Rnq1-EcoRV | 5'GGGGATATCATGGATACGGATAAGTTAATCTCAGAGG |
| 0489 | 3' Rnq1-SalI | 5'CCCGTCGACTCAGTAGCGGTTCTGGTTGCCG |
| 0491 | 3' Rnq1 terminator-XhoI | 5'CCCCTCGAGGCAACACATACCTTACAAAGC |
| 1429 | 5’ TPI1prom-SacI | 5’GCGGAGCTCCTACGTATGGTCATTTCTTC |
| 1430 | 3’ TPI1prom-XbaI | 5’GCGTCTAGATTTTAGTTTATGTATGTG |
| 1244 | 5' Rnq1-A1 | 5'CAAATGTCAACTATTGAATCAGCAGCTGCAGCAGCCGCAGCAGCCGCCATGGACAACCGTAGT |
| 1245 | 3' Rnq1-A1 | 5'CACCACTACGGTTGTCCATGGCGGCTGCTGCGGCTGCTGCAGCTGCTGATTCAATAGTTGACATTTG |
| 1472 | 5’ Rnq1-A2 | 5'GTGGTGGTGCAGCTGCTGCGGCCGCTGCAGCGGCAGACTCTAAGGGTTC |
| 1473 | 3’ Rnq1-A2 | 5'GAGTCTGCCGCTGCAGCGGCCGCAGCAGCTGCACCACCACCAGCAGCACG |
| 1442 | 5’ Rnq1-A3 | 5'GCAGCAGCAGCTGCGGCAGCCGCAGCGGCGGCACACTCATCAAATAAAGG |
| 1443 | 3’ Rnq1-A3 | 5'TGCCGCCGCTGCGGCTGCCGCAGCTGCTGCTGCTAGTTGCGTTTGGGAAG |
| 1426 | 5’ Rnq1-A4 | 5’CTTCTAACAGAGGGTTTGCCGCAGCGGCTGCCGCGGCAGCGGCAAGTGGTTCTGGCGGC |
| 1427 | 3’ Rnq1-A4 | 5’GCCGCCAGAACCACTTGCCGCTGCCGCGGCAGCCGCTGCGGCAAACCCTCTGTTAGAAG |
| 1474 | 5’ Rnq1-A5 | 5'GTGCTTCCGCCGCGGCTGCCGCGGCTGCTGCAGCCGCTAAGTCAGGTAACAATTCCC |
| 1475 | 3’ Rnq1-A5 | 5'CTTAGCGGCTGCAGCAGCCGCGGCAGCCGCGGCGGAAGCACCCATACTTTG |
| 1444 | 5’ Rnq1-A6 | 5'GCTGCTGCTGCTGCGGCGGCTGCGGCTGCAGCTGCCATGAATTCCAACAAC |
| 1445 | 3’ Rnq1-A6 | 5'GGCAGCTGCAGCCGCAGCCGCCGCAGCAGCAGCAGCACCTTGACCTTGACCTTGTCCTTGACCTTGACC |
| 1547 | 5’ Rnq1-A7 | 5’GCTCCGGTGGTTCCGCCGCTGCAGCAGCGGCTGCTGCGGCAGCCGCTGCTATGCATTCCAATAATAATC |
| 1548 | 3’ Rnq1-A7 | 5’GATTATTATTGGAATGCATAGCAGCGGCTGCCGCAGCAGCCGCTGCTGCAGCGGCGGAACCACCGGAGC |
| 1478 | 5’ Rnq1-A8 | 5'GCTGCTGCCGCAGCGGCCGCCGCGGCTGCAGCTGCCTTAGGTGGTGGACAAACTC |
| 1479 | 3’ Rnq1-A8 | 5'CTAAGGCAGCTGCAGCCGCGGCGGCCGCTGCGGCAGCAGCACCACCAGATTGTCCC |
| 1246 | 5' Rnq1-A9 | 5'GGTGGTGGACAAACTCAATCCGCCGCAGCGGCAGCCGCTCAACAAGGCCAAAACAACCAGCAGCAA |
| 1247 | 3' Rnq1-A9 | 5'TTGCTGCTGGTTGTTTTGGCCTTGTTGAGCGGCTGCCGCTGCGGCGGATTGAGTTTGTCCACCACC |
| 1480 | 5’ Rnq1-A10 | 5'GTGCAGCCGCAGCTGCGGCTGCCGCGGCAGCTGCCGCCCTGGGCAATAACTCCAATTC |
| 1481 | 3’ Rnq1-A10 | 5'CAGGGCGGCAGCTGCCGCGGCAGCCGCAGCTGCGGCTGCACTGGAGTGGCCTTGTTGC |
| 1498 | 3’ Rnq1-A11 | 5’GAGCAGCGGCGGCAGCAGCAGCGGCGGCTTCGTGCTGTCCATTGGAG |
| 1525 | 5’ Rnq1-A11 | 5’CGAAGCCGCCGCTGCTGCTGCCGCCGCTGCTCAACAGAACAATAACGGC |
| 0040 | 3'-XhoI-RNQ | 5'CCGCTCGAGTCATCAGTAGCGGTTCTGGTTGCC |
| 1626 | 5’Rnq1-153-405-HindIII | 5’GCGAAGCTTATGCAAGGTCAGGGACAAGG |
| 1436 | 5’Rnq1-del131 | 5'GCGGATATCATGAGTATGGGTGCTTCCGGC |
